# Mechanism underlying circRNA dysregulation in the TME of digestive system cancer

**DOI:** 10.3389/fimmu.2022.951561

**Published:** 2022-09-27

**Authors:** Zeyu Wu, Xiao Yu, Shuijun Zhang, Yuting He, Wenzhi Guo

**Affiliations:** ^1^ Department of Hepatobiliary and Pancreatic Surgery, The First Affiliated Hospital of Zhengzhou University, Zhengzhou, China; ^2^ Key Laboratory of Hepatobiliary and Pancreatic Surgery and Digestive Organ Transplantation of Henan Province, The First Affiliated Hospital of Zhengzhou University, Zhengzhou, China; ^3^ Open and Key Laboratory of Hepatobiliary & Pancreatic Surgery and Digestive Organ Transplantation at Henan Universities, Zhengzhou, China; ^4^ Henan Key Laboratory of Digestive Organ Transplantation, The First Affiliated Hospital of Zhengzhou University, Zhengzhou, China

**Keywords:** circular RNAs, tumor microenvironment, molecular mechanism, digestive system cancer, chemotherapy resistance

## Abstract

Circular RNAs (circRNAs) are a new series of noncoding RNAs (ncRNAs) that have been reported to be expressed in eukaryotic cells and have a variety of biological functions in the regulation of cancer pathogenesis and progression. The TME, as a microscopic ecological environment, consists of a variety of cells, including tumor cells, immune cells and other normal cells, ECM and a large number of signaling molecules. The crosstalk between circRNAs and the TME plays a complicated role in affecting the malignant behaviors of digestive system cancers. Herein, we summarize the mechanisms underlying aberrant circRNA expression in the TME of the digestive system cancers, including immune surveillance, angiogenesis, EMT, and ECM remodelling. The regulation of the TME by circRNA is expected to be a new therapeutic method.

## Introduction

**Figure 1:**
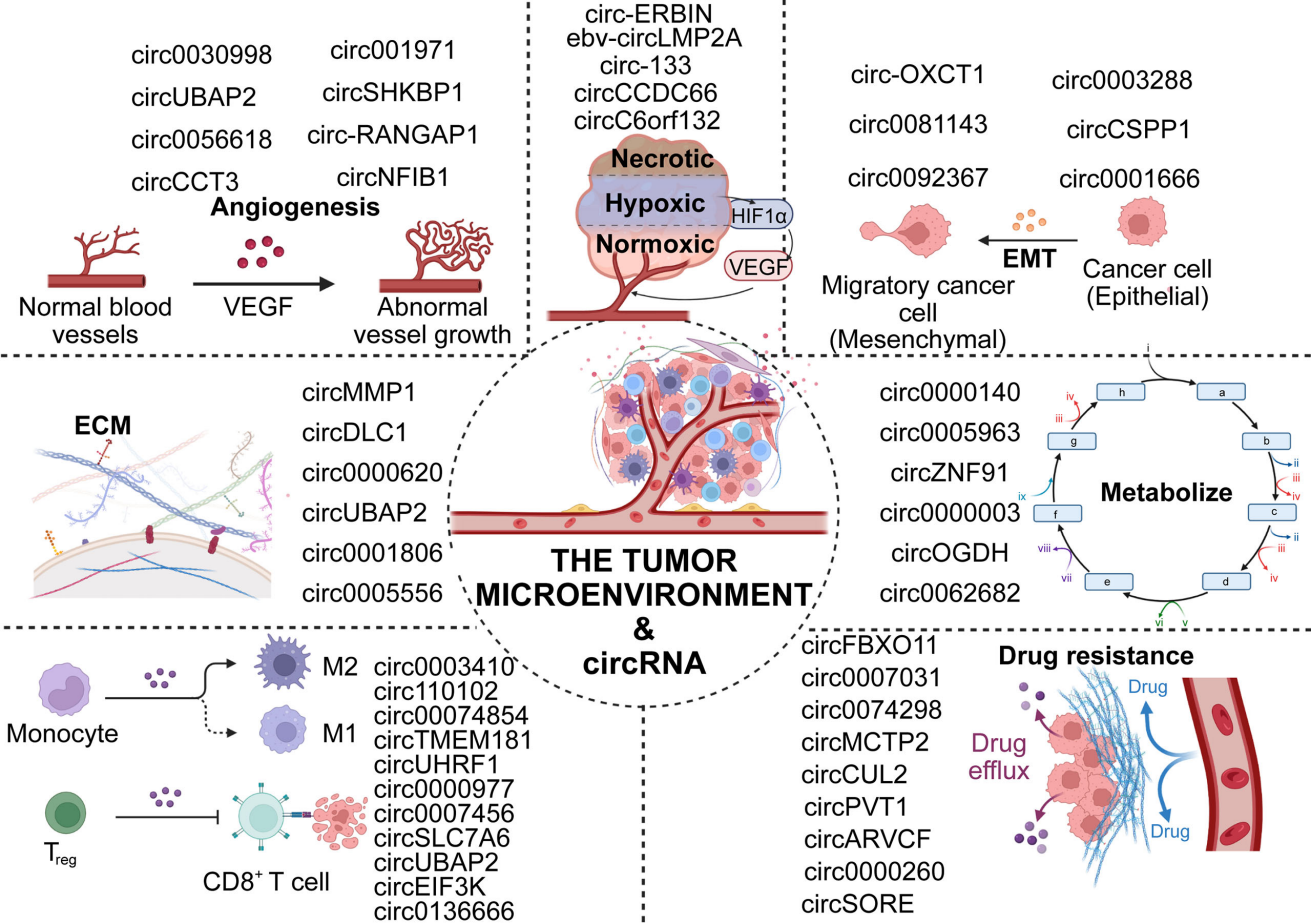
Association of circRNAs with the TME.

Cancer of the digestive system (DSC) has the highest mortality rate among invasive cancers worldwide. Although current treatments, including surgery, radiotherapy, and immunotherapy are improving, the average survival time of patients with advanced DSC remains low due to the cryptic, rapid, and aggressive nature of early symptoms (1–4). In recent years, numerous studies have reported that the tumor microenvironment (TME) plays a critical role in the genesis and development of digestive tumors (5–8). The TME represents the immediate ecological environment for tumor growth and is composed of multiple cell types that collectively participate in complex regulation (9, 10). These cellular interactions are conducive to tumor progression, immune escape, angiogenesis, and metastasis (11), and play crucial roles in the chemoresistance of cancer. (**Figure 1**)

Recently, many circRNAs in eukaryotic cells have been characterized by high-throughput RNA sequencing and new bioinformatics algorithms, and have crucial roles in different types of cancers (12–15). In terms of microstructure, circRNAs have been found to be ncRNAs with covalently closed structures, transcribed by RNA polymerase II, without 5’-3’ polar or polyadenine tails (16, 17). This covalently closed circular structure makes circRNA less susceptible to exonuclease digestion than linear RNA (15, 18, 19). Therefore, aberrant expression of circRNA may accumulate in cells, which can lead to cancer progression (20). CircRNAs mainly participate in and regulate the progression of DSC by influencing immune cells, energy metabolism, signal transduction, angiogenesis, and lymphatic duct formation in the TME (18, 21, 22). In addition, numerous studies have shown that circRNAs play a key role in human DSCs as diagnostic markers, prognostic targets, and therapeutic targets (9, 23–26).

At present, the crosstalk between circRNAs and various components in the TME has attracted great attention. The role of circRNAs in the TME may become another emerging direction for cancer treatment. Recent studies on circRNAs mainly focus on their impact on cancer biological behavior, but lack a systematic summary of the tumor microenvironment, especially in digestive system tumors.

In this review, we summarize the clinical features and biological functions of DSCs affected by circRNAs and the regulatory mechanisms in the TME. CircRNAs will be a potentially useful tool for the diagnosis and therapeutic targeting of DSC. Finally, we also discussed the messenger role of exosomal circRNAs in the TME of digestive system cancers, and revealed the vital role of exosomal circRNAs in signal transmission.

## Biogenesis, regulation and degradation of CircRNAs

**Figure 2:**
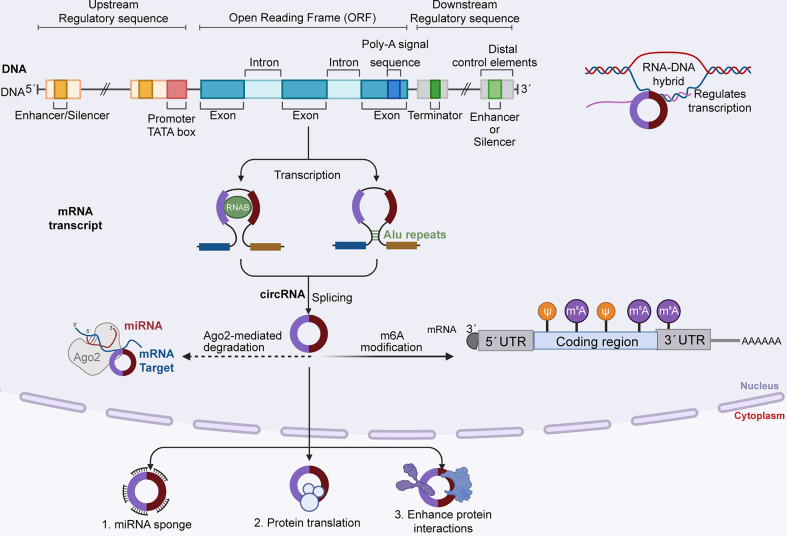
DNA generates circRNA precursors by transcribing local exons, and mature circRNAs are formed by reverse splicing. CircRNA degrades in two ways: 1) RNA degradation mediated by AGO2 protein, in which MiRNA binds to circRNA first and then guides AGO2 to perform dependent cleavage; and 2) m6A modification, and CircRNA can be cleaved by endoribonuclease after m6A modification. Mature circRNA functions in three ways: as a miRNA sponge to regulate downstream targets, translate proteins, and enhance protein reactions.

It was once thought that the translation mechanism of eukaryotic cells could not translate circRNAs due to the circRNA’s unique ring structure (27). However, the discovery of internal ribosome entry sites and m6A completely overturned this thinking (28–32). These mechanisms promote the independent initiation of translation at the 5’ end of circRNA and enable circRNA to exert a strong influence on translation control through its sponge function to form a new mRNA family (33–36) (**Figure 2**).

Most current studies have reported that circRNAs are the products of back-splicing of the precursor mRNA of the exon, and its downstream 5’ splice site is connected with the upstream 3’ splice site by a 3’-5’ phosphodiester bond at the junction site (37, 38). The formation of circRNA mainly includes three back-splicing models: exon-skipping, intron pairing, and RNA-binding protein interactions, and the three models contain different mechanisms (39). The first model is exon hopping, which results in the loss of one or more exons of a mature mRNA. In this model, the lariat-driven circularization proceeds as two nonadjacent exons join together, finally producing an mRNA with skipped exons, a circular RNA transcript and a lariat structure. This circularization can achieve a more efficient circle production (40).

The second model is intron pairing-driven circularization. In this biogenesis mechanism, two introns flanking the pre-mRNA exon/exon have one structure capable of interlinking. Flanking introns are close to each other, forming a secondary conformation that allows reverse splicing of the splice site. Most intron pairing patterns are promoted by ALU repeats. The longer the intron length is, the greater the chance that they will have more ALU elements, thereby enhancing exon circularization.

Third, the main mechanism of this mode is through RNA-binding proteins (RBPs). RBPs are able to bind to pre-mRNA and link flanking introns together, this process is induced by protein dimerization, which creates an RNA loop.

The expression level of homeostatic circRNAs can be regulated at three stages. First, the biogenesis of circRNA begins with the transcription and binding of Pol II to the pre-mRNA that produces circRNA (41); second, cis and trans regulators can further affect the efficiency of back-splicing catalyzed by spliceosomal mechanisms (42, 43); and finally, circRNA turnover also plays an important role in expression levels (44).

At present, circRNAs mainly regulate downstream targets through high expression in cancer, so it is critical to understand the degradation and inactivation of circRNAs for future targeted therapy (45). Three main types of nucleases are involved in RNA decay: 5’ exonuclease, 3’ exonuclease, and endonuclease, which cleave RNA from the inside. However, due to the unique closed-loop structure of circRNA, the degradation of circRNA should be mediated by nicking endonucleases (42, 46, 47). The first mode of endonuclease induced circRNA degradation is mediated by Ago2, which relies on endogenous guide RNAs, such as miRNAs, to execute its function (48). miRNA first binds to circRNA in base pairing and directs Ago2-dependent cleavage. For instance, circAGO2 interacts with HuR, resulting in reduced AGO2 binding and the promotion of tumorigenesis and invasiveness (49–51). Moreover, miR1224 splices circFilip1L to regulate chronic inflammatory pain in an Ago2-dependent manner by targeting Ubr5 (52). However, Ago2-dependent circRNA degradation does not work for circRNAs that have no specific miRNA target (53). It is at this point that the role of m6A modification comes into focus. The important role of m6A modification in circRNA regulation has been reported in many recent articles (54–56). m6A modified circRNA is also cleaved by endoribonuclease *via* the YTHDF2-HRSP12-RNase P/MRP axis (57); however, the number of circRNA degradations mediated by m6A modification reported thus far remains limited, and further studies are needed in this area. In addition to the above, adenosine deaminase 1 acting on RNA (ADAR1), as a dsRNA-binding protein, can inhibit the intron pairing process of circRNA formation by reducing the pairing activity of the ALU repeat series, thus preventing the formation of circRNA. On the other hand, ADAR1 can interrupt miRNA processing, thus regulating the formation of circRNA. A deeper understanding of circRNA biosynthesis, regulation and degradation can facilitate further targeted therapies against cancers caused by circRNA dysregulation.

## Biological functions of circRNAs

Current studies have shown that circRNAs perform their biological functions in four main ways: miRNA sponge, transcriptional regulation, coding for proteins and peptides, functions with RNA-binding proteins (54, 58, 59).

## miRNA sponge

Most of circRNAs involved in this review can regulate downstream targets by acting as miRNA sponges to affect the TME in digestive system cancers. Although circRNA has a unique closed-loop structure, it still contains miRNA binding sites, which endows it with potential as a miRNA sponge (60–62). This property suggests that circRNAs can inhibit the activity of mature miRNAs, increase the level of endogenous targets, and inhibit miRNAs to regulate the expression of downstream genes (63, 64). For example, CIRS-7, which contains more than 70 miRNA seed regions, is considered a ceRNA and plays an important role in a variety of cancers (65, 66). In esophageal cancer, CIRS-7 upregulates the expression of HOXB13 by sponging miR-7, thereby promoting the proliferation, invasion and metastasis of tumor cells (67). In gastric cancer, CIRS-7 promotes cancer cell proliferation and invasion through the miR-7/PTEN/PI3K/AKT pathway (68). These effects are achieved by CIRS-7 acting as a molecular sponge.

## CircRNA functions with RNA-binding proteins

In addition to sponging miRNAs, circRNAs also bind to different RBPS with different potential roles: to inhibit protein function (protein bait), to promote protein complex formation and to allow interactions between different proteins (69). First the protein bait, CircMBl (70), as a highly expressed and evolutionarily conserved circRNA, contains multiple binding sites for the MBL protein as well as part of the MBL open reading frame, and has been shown to be translatable (71). There was a good correlation between circMbl and MBL levels, suggesting that circMbl biosynthesis could be adjusted according to MBL protein levels (72). Second, circRNA forms a complex with protein. As a circRNA closely related to cell cycle progression, circ-foxo3 binds to cell division protein kinase 2 (CDK2) and cyclin-dependent kinase inhibitor 1 (OR P21) to form ternary complex (73). CDK2 can promote the cell cycle, while P21 inhibits cell cycle progression, and eventually the formation of this ternary complex prevents CDK2 function and blocks cell cycle progression.

## Coding for proteins and peptides

Although circRNAs were originally defined as noncoding RNAs, they still have the ability to encode proteins, depending on their specific structures, such as the presence of internal ribosome entry site (IRES) (74),and N6-methyladenosine (m6A)-mediated initiation (75, 76). Even in the absence of the 5’ cap and related cap-binding protein factors, circRNAs can recruit ribosomal 40S subunits to initiate translation *via* specialized sequences in the 5’ noncoding region and then produce small proteins and micropeptides (16).

## Transcriptional regulation

In addition, circRNA also plays a role in transcriptional regulation, and by regulating two key steps, circRNA can enhance transcription at the transcriptional level (77). The first step is the initiation step, and circRNAs are only involved in the formation of preinitiation complexes. The last step is during the elongation phase, CircRNAs accumulate at their site of transcription and increase parental gene transcription elongation by interacting with RNA polymerase II (78, 79). Additionally, the biogenesis of circRNAs *via* exon skipping can be seen as a passive function of circular transcripts (41).

## Aberrant circRNA expression regulates the clinical features and cell biological functions of DSCs

A range of abnormally expressed circRNAs have been proven to be associated with the progression of DSC, which may lead to the development of a new understanding of the clinical application of circRNAs. In this section, we summarize the association between abnormal circRNA expression and biological functions (**Table 1**) and clinicopathological features (**Table 2**).

**Table 1:** Cytological function and molecular axis of circRNA in various digestive system tumors.

Cancer type	CircRNA	Role in cancer progression	Cell Function	Axis	Refs.
HCC	circ0003410	promotor	promote HCC cell proliferation and migration	circ0003410/miR-139-3p/CCL5 Axis	(80)
HCC	Circ0110102	suppressor	suppress HCC cell growth, migration, and invasiveness	circ0110102/miR-580-5p/PPARα/CCL2	(83)
HCC	circ0074854	promotor	promote HCC cell proliferation and inhibit apoptosis; knockdown of circ00074854 suppress migration, invasion and EMT	/	(91)
HCC	circTMEM181	promotor	CD39 attenuate the immune response signal stimulated by eATP in tumor microenvironment	circTMEM181/miR-488-3p/CD39	(89)
HCC	circUHRF1	promotor	immune evasion	circUHRF1/miR-449c-5p/TIM-3	(90)
HCC	circ0007456	suppressor	increase NK cell sensitivity to tumor cells	circ0007456/miR-6852-3p/ICAM-1	(85)
HCC	circUBAP2	promotor	promote HCC cell migration	circUBAP2/miR-4756/IFIT1/IFIT3 axis	(87)
HCC	circDLC1	suppressor	overexpression of circDLC1 inhibits the proliferation and motility of HCC cells in vitro and in vivo	circDLC1/HUR/MMP1	(86)
HCC	circUBAP2	promotor	promote the migration, invasion, and proliferation of HCC cells	circUBAP2/miR-194-3p/MMP9	(81)
HCC	circ0001806	promotor	knockdown circ0001806 suppressed the proliferation, migration, and invasion of HCC cells	circ0001806/miR-193a-5p/MMP16	(92)
HCC	circ_0003288	promotor	promote EMT, migration, and invasion of HCC	circ0003288/miR-145/PD-L1	(88)
HCC	circFBXO11	promotor	circFBXO11 overexpression alleviated the cycle arrest and apoptosis, circFBXO11 knockdown repressed the tumor growth in vivo	circFBXO11/miR-605/FOXO3/ABCB1	(82)
HCC	circSORE	promotor	circRNASORE knockdown increased apoptosis in sorafenib-resistant cells	circSORE/miR-103a-2-5p/miR-660-3p/β-catenin signaling	(84)
GC	Circ0000620	promotor	circ0000620 knockdown reduced cell viability, colony formation, migration, invasion and tube formation capacity	circ0000620/miR-671-5p/MMP2	(102)
GC	circ0005556	promotor	circ0005556 knockdown can inhibit the migration and invasion, and arrest the cell cycle in the G2/M phase	circ0005556/miR-4270/MMP19	(109)
GC	circSHKBP1	promotor	promote GC cell proliferation, migration and invasion	circSHKBP1/miR-582-3p//HUR/VEGF	(99)
GC	circRANGAP1	promotor	circRANGAP1 silencing suppressed tumor growth and metastasis in vivo, decreased GC cell invasion and migration in vitro	circRANGAP1/miR-877-3p /VEGFA	(100)
GC	ebv-circLMP2A	promotor	promoted hypoxia-induced tube formation, migration, and angiogenesis	ebv-circLMP2A/KHSRP/VHL/HIF1α/VEGFA	(107)
GC	circC6orf132	promotor	promote cell proliferation, migration, and invasion of gastric cancer cells	circC6orf132/miR-873-5p/PRKAA1	(110)
GC	circ-OXCT1	suppressor	circ-OXCT1 overexpression suppressed cell migration and invasion,	circOXCT1/miR-136/SMAD4	(108)
GC	circ0081143	promotor	migration, invasion, and EMT	circ0081143/miR-497-5p/EGFR	(111
GC	circMCTP2	suppressor	promote apoptosis of CDDP resistant GC cells in response to CDDP treatment, and reducing cell proliferation	circMCTP2/miR-99a-5p/MTMR3	(106)
GC	circCUL2	suppressor	overexpression of circCUL2 inhibited autophagy, and inhibited cell proliferation, migration and invasion	circCUL2/miR-142-3p/ROCK2	(101)
GC	circPVT1	promotor	circPVT1 knockdown repressed DDP resistance in DDP-resistant GC cells by inducing apoptosis and inhibiting autophagy	circPVT1/miR-30A-5p /YAP1	(103)
GC	circARVCF	promotor	promote cell invasion and metastasis, inhibit apoptosis	circARVCF/miR-1205/FGFR1	(104)
GC	circ0000260	promotor	circ0000260 knockdown inhibited proliferation, migration, invasion and adhesion of CDDP-resistant GAC cells	circ0000260/miR-129-5p /MMP11	(105)
PC	circ0000977	promotor	HIF1A mediated immune escape of PC cells, ADAM10 leaded to low reactivity of NK cells	circ0000977/miR-153/HIF-1A/ADAM10	(184)
PC	circZNF91	promotor	increase tumor size	circZNF91/miR-23b-3p/ HIF-1α	(98)
PC	circCCT3	promotor	promote the migration, invasion of PC cells, and tumor volume and weight	circCCT3/miR-613/VEGF/VEFGR2	(93)
PC	circNFIB1	suppressor	overexpression of circNFIB1 inhibited lymphangiogenesis of PDAC in vitro, inhibited LN metastasis of PDAC in vivo	circNFIB1/miR-486-5p/PIK3R1/VEGF-C	(96)
PC	circ0001666	promotor	knockdown of circ_0001666 inhibited the proliferation of PC cells, represses EMT in PC	circ0001666/miR-1251/SOX4	(94)
PC	circ0092367	suppressor	overexpression of circ0092367 inhibited xenograft tumor growth, cell invasion, EMT, and gemcitabine resistance	circ0092367/miR-1206/ ESRP1	(97)
PC	circ0074298	promotor	downregulation of circ0074298 significantly inhibited cell proliferation, migration, invasion, colony formation and promoted cell cycle arrest, apoptosis and chemo-resistance of pancreatic cancer in vitro and vivo	circ0074298/miR-519/SMOC	(95)
CRC	circSLC7A6	promotor	promote cell proliferation and invasion, and decreased apoptosis	/	(117)
CRC	circEIF3K	promotor	promote cell proliferation, enhance cell colony formation	circEIF3K/miR-214/PD-L1 axis	(116)
CRC	circ0136666	promotor	Treg-mediated immune escape	circ0136666/miR-497/PD-L1	(126)
CRC	circMMP1	promotor	circMMP1 knockdown inhibits the growth and metastasis of CRC in vivo, suppresses the proliferation and invasion of CRC cell in vitro	circMMP1/miR-1238/MMP1/MMP2/MMP9	(123)
CRC	circ0005963	promotor	enhance glycolysis and drug resistance to increase the size of drug-resistant tumors in vivo	circ0005963/miR-122/PKM2	(119)
CRC	circ0062682	promotor	enhance the proliferation and colony formation of CRC cells	circ0062682/miR-940/PHGDH	(120)
CRC	circMYH9	promotor	promoted cell cycle and increased cell cycle proteins	circMYH9/p53	(114)
CRC	circ0030998	promotor	promoted tumor proliferation and angiogenesis in vitro	circ0030998/miR-567/VEGFA	(115)
CRC	circUBAP2	promotor	circUBAP2 knockdown inhibited CRC cell migratory and invasive abilities	circUBAP2/miR-199a/VEGFA	(121)
CRC	circ0056618	promotor	promoted cell proliferation, migration and angiogenesis	circ0056618/CXCR4/VEGF-A	(124)
CRC	circ-ERBIN	promotor	accelerate the proliferation, migration, invasion and metastasis of CRC cells in vitro and in vivo	circ-Erbin/miR-125a-5p-5p/miR-138-5p/4EBP-1/HIF-1α	(122)
CRC	circ-133	promotor	increased cell migration capacity	circ-133/GEF-H1/RhoA	(112)
CRC	circCCDC66	promotor	circCCDC66-knockdown reduced viability, migration and invasion, and enhanced the apoptosis of hypoxia-exposed CRC cells	circCCDC66/miR‑3140/autophagy	(125)
CRC	circCSPP1	promotor	circCSPP1 promoted CRC cell migration and invasion in vitro, promoted tumor cell liver metastasis in vivo, promoting the progression of EMT	circCSPP1/miR-193a-5p/COL1A1	(113)
CRC	circ0007031	promotor	circ0007031 knockdown repressed CRC cell proliferation, migration and invasion and enhanced 5-FU sensitivity	circ0007031/miR-133b/ABCC5	(118)
ESCC	circOGDH	promotor	accelerated proliferation, metastasis, and invasion of ESCC cells	circ-OGDH/miR-615-5p/PDX1	(127)
OSCC	circ0000140	suppressor	overexpression of circ0000140 blocked the proliferation, migration, and invasion of OSCC cells	circ0000140/miR-182-5p/CDC73	(128)
TSCC	circ0000003	promotor	circ0000003 knockdown significantly inhibited cell invasion and migration, overexpression of circ0000003 promoted cell proliferation	circ0000003/miR‑330‑3p/GLS axis	(129)

**Table 2:** Correlation of circRNAs with clinical features in digestive system tumors.

Cancer type	CircRNA	Expression	Clinical features	Refs.
HCC	circ0003410	overexpression	tumor size	(80)
HCC	circ110102	low-expression	survival rate	(83)
HCC	circ00074854	overexpression	/	(91)
HCC	circTMEM181	overexpression	anti-PD1 therapy resistance, early recurrence, microvascular invasion	(89)
HCC	circUHRF1	overexpression	increase tumor size and microvascular invasion, reduce overall survival	(90)
HCC	circ0007456	low-expression	primary tumor stage, lymph node metastasis	(85)
HCC	circUBAP2	overexpression	promote tumor migration and metastasis	(87)
HCC	circDLC1	low-expression	advanced tumor stage, TNM stage and BCLC stage, microvascular invasion, macrovascular invasion	(86)
HCC	circUBAP2	overexpression	tumor size and high tumor recurrence rate	(81)
HCC	circ0001806	overexpression	/	(92)
HCC	circ0003288	overexpression	migration and invasion	(88)
HCC	circFBXO11	overexpression	tumor size	(82)
HCC	circSORE	overexpression	recurrence free survival and overall survival	(84)
GC	circ0000620	overexpression	overall survival	(102)
GC	circ0005556	overexpression	/	(109)
GC	circSHKBP1	overexpression	advanced pathological staging and poor survival	(99)
GC	circRANGAP1	overexpression	advanced TNM stage, lymph node metastasis, and poor survival	(100)
GC	ebv-circLMP2A	overexpression	tumor invasion and metastasis	(107)
GC	circC6orf132	overexpression	/	(110)
GC	circOXCT1	low-expression	lymphatic node metastasis, pathological stages, 5-year overall survival	(108)
GC	circ0081143	overexpression	/	(111)
GC	circMCTP2	low-expression	CDDP chemosensitivity, tumor size, TNM stage	(106)
GC	circCUL2	low-expression	Cisplatin resistance, late-stage GC (stage III+IV), lymph node metastasis, poor differentiation and poor overall survival	(101)
GC	circPVT1	overexpression	CIS resistance	(103)
GC	circARVCF	overexpression	DDP resistance	(104)
GC	circ0000260	overexpression	CDDP resistance	(105)
PC	circ0000977	overexpression	/	(184)
PC	circZNF91	overexpression	promoted chemoresistance, overall survival	(98)
PC	circCCT3	overexpression	Vascular invasion, peritoneal metastasis, lymph node metastasis and clinical progression	(93)
PC	circNFIB1	low-expression	lymphatic metastasis and high pathological TMN stage	(96)
PC	circ0001666	overexpression	overall survival, lymphatic metastasis	(94)
PC	circ0092367	low-expression	advanced tumor stage, lymph node metastasis	(97)
PC	circ0074298	overexpression	tumor diameter, lymphatic metastasis, and pathological grade	(95)
CRC	circSLC7A6	overexpression	overall survival, advanced stages (Stage III and IV)	([Bibr B117]117)
CRC	circEIF3K	overexpression	overall survival, advanced stages	(116)
CRC	circ0136666	overexpression	/	(126)
CRC	circMMP1	overexpression	/	(123)
CRC	circ0005963	overexpression	Enhancing oxaliplatin resistance	(119)
CRC	circ0062682	overexpression	Tumor stage	(120)
CRC	circMYH9	overexpression	overall survival, tumor size, distant metastasis, lymph node metastasis, TNM stage, and p53 status	(114)
CRC	circ0030998	overexpression	Lymph node metastasis and TNM stage, shorter survival	(115)
CRC	circUBAP2	overexpression	/	(121)
CRC	circ0056618	overexpression	/	(124)
CRC	circ-ERBIN	overexpression	/	(122)
CRC	circ133	overexpression	metastasis	(112)
CRC	circCCDC66	overexpression	/	(125)
CRC	circCSPP1	overexpression	overall survival, metastasis	(113)
CRC	circ0007031	overexpression	anti-5-fu chemotherapy	(118)
ESCC	circOGDH	overexpression	promote tumor growth	(127)
OSCC	circ0000140	low-expression	lymph node metastasis	(128)
TSCC	circ0000003	overexpression	advanced TNM stage and increased tumor size	(129)

## Hepatocellular carcinoma

**Figure 3:**
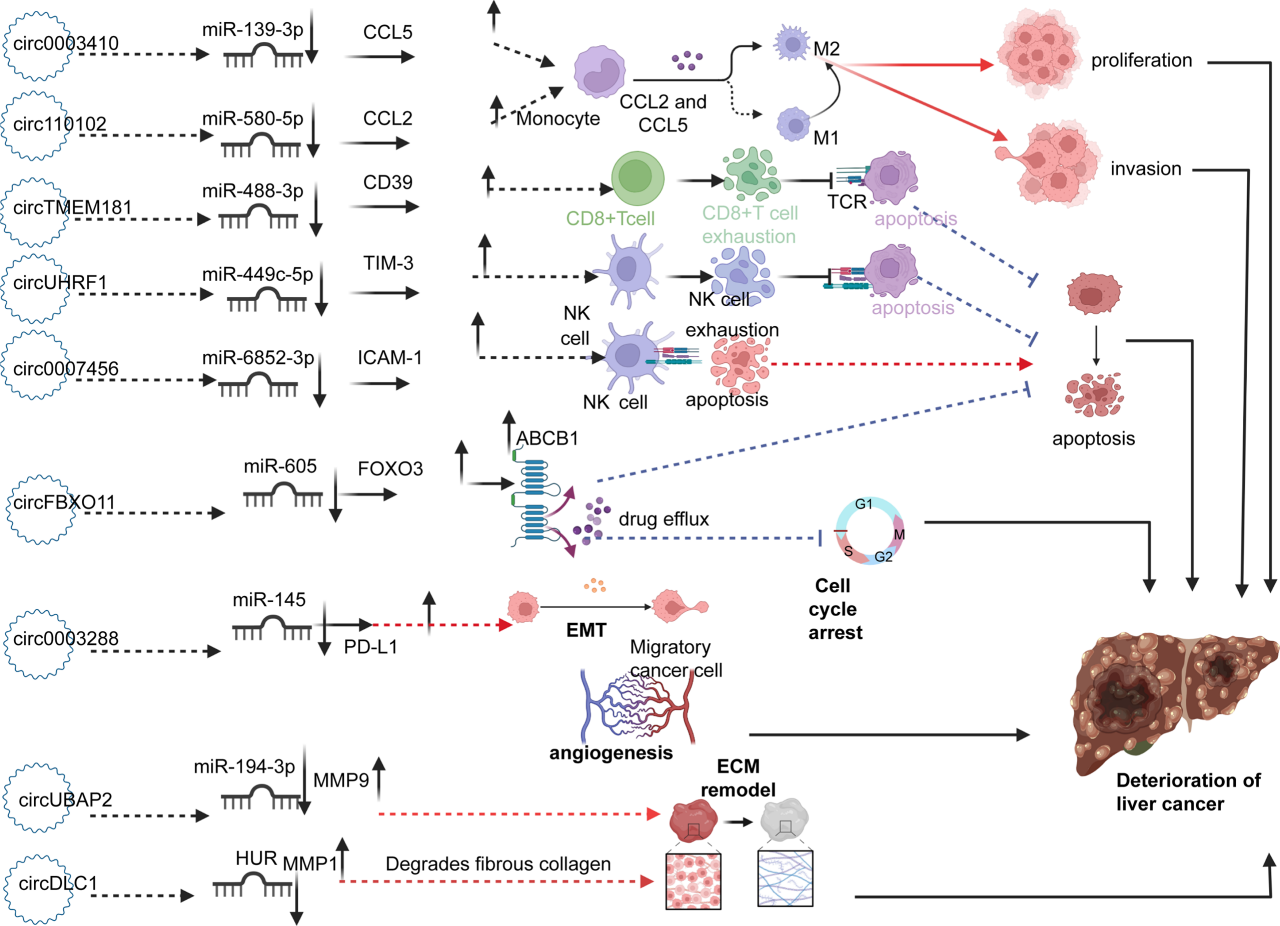
Circ0003410 and circ110102 upregulate CCL5 and CCL2 by sponging miR-139-3p and miR-580-5p, respectively, thereby promoting the polarization of M2 macrophages and promoting cell proliferation and invasion. CircTMEM181 and circUHRF1 promote depletion of CD8+T and NK cells through the miR-488-3p/CD39 and miR-499-5p/TIM-3 axis, thus inhibiting apoptosis of cancer cells. Circ0007456 upregulates CAM-1 by sponging miR-6852-3p, which increases the lethality of NK cells to cancer cells and promotes tumor cell apoptosis. CircFBXO11 increases the expression of ABCB1 protein on the surface of cancer cells by upregulating FOXO3 and enhancing drug resistance. Circ0003288 promotes EMT through Mir-145/PDL1. ECM remodeling is mediated by the upregulation of MMP1 and MMP9 by circUBAP2 and circDLC1, thereby promoting metastasis. The role of circRNAs above promotes the occurrence and deterioration of HCC.

In patients with HCC, a series of circRNAs have been demonstrated to be closely related to the tumorigenesis and development of HCC. (**Figure 3**) For example, high expression of circ0003410, circUBAP2, and circFBXO11 was positively related to tumor size (80–82). Low expression of circ110102 and high expression of circSORE were associated with low survival rates, of which circSORE was also negatively correlated with recurrence-free survival (83, 84). As tumor suppressor factors, low expression of circ0007456 and circDLC1 is associated with poor prognosis, tumor stage, lymph node metastasis stage, microvascular invasion, and macrovascular invasion (85, 86). Furthermore, upregulated circUBAP2 and circ0003288 were positively correlated with distant metastasis and invasion (87, 88). High expression of circTMEM181 and circUHRF1 enhances anti-PD1 resistance, causing reduced overall survival, early recurrence, and a high rate of microvascular invasion (89, 90). In terms of biological function, high expression of circ0003410, circ00074854, and circDLC1 could promote HCC cell proliferation and motility *in vivo* and *in vitro (* 80, 86, 91). CircUBAP2 and circ0001806, as tumor promoters, promote distant metastasis of cancer cells *in vivo* by enhancing the migration and invasion abilities of cells (87, 92). Circ110102, as a tumor suppressor, can attenuate invasion and thus weaken the metastatic ability of tumor cells (83). CircFBXO11 and circSORE promote malignant proliferation by inhibiting apoptosis (82, 84). CircTMEM181 and circUHRF1 attenuate the immune response signal to promote the immune escape of HCC cells and prevent the killing ability of immune cells (89, 90). In contrast, circ0007456 has been shown to increase the sensitivity of natural killer (NK) cells to tumor cells and thus enhance the killing effect of immune cells (85).

## Pancreatic cancer

Given the highly metastatic nature of pancreatic cancer cells, distant metastasis, especially lymphatic metastasis, occurs in the early stage of pancreatic cancer. Relevant studies have shown that high expression of circ0074298, circCCT3, and circ0001666 in pancreatic cancer is closely related to lymph node metastasis (93–95), while low expression of circNFIB1 and circ0092367 is related to advanced TNM stage, lymph node metastasis, and overall survival (96, 97). High expression of circZNF91 promotes chemotherapy resistance in pancreatic cancer cells and is inversely proportional to overall survival (98). In terms of cellular activities, downregulation of circ0074298 has been shown to significantly inhibit the malignant phenotype and promote apoptosis and chemotherapy resistance of pancreatic cancer *in vitro* and *in vivo (* 95). High expression levels of circZNF91 and circCCT3 promote the migration and invasion of pancreatic cancer cells, and increase tumor volume and weight *in vivo (* 93, 98). Overexpression of circNFIB1 inhibits lymphangiogenesis of PDAC *in vitro* and LN metastasis of PDAC *in vivo (* 96). As a tumor suppressor, overexpression of circ0092367 inhibits xenograft tumor growth, cell invasion, epithelial-mesenchymal transformation (EMT), and gemcitabine resistance (97).

## Gastric carcinoma

In GC, high expression of circSHKBP1 and circRANGAP1 plays a role in promoting cancer, which is closely associated with lymph node metastasis and advanced TNM staging. Low expression of circCUL2, a tumor suppressor, is positively correlated with the above cancer characteristics (99–101). Upregulation of circ0000620 was shown to be negatively correlated with overall survival of GC (102). In terms of drug resistance, upregulated circPVT1 mediates CIS resistance, and high expression of circARVCF, circ0000260 and low expression of circMCTP2 are closely related to CDDP resistance, thus reducing the efficacy of chemotherapy (103–106). Moreover, Ebv-circLMP2A has been shown to promote the invasion and metastasis of GC cells, while high expression of circOXCT1 inhibits lymph node metastasis and pathological stage, which is positively associated with the 5-year survival rate (107, 108). In terms of cellular function, circ0000620 and ebv-circLMP2A enhance the tube formation capacity and angiogenesis (102, 107), and upregulation of circ0005556, circSHKBP1, circ-RANGAP1, and circc6orf132 increase the invasion and migration ability (99, 100, 109, 110). CircOXCT1 overexpression was reported to suppress cell migration and invasion, and circ0081143 promotes GC cell invasion and metastasis by promoting EMT (108, 111). CircMCTP2 overexpression and circARVCF knockdown promote apoptosis of CDDP-resistant GC cells, thereby reducing cell proliferation (104, 106). Overexpression of circCUL2 and circPVT1 knockdown have been shown to inhibit autophagy and prevent chemotherapy resistance (101, 103).

## Colorectal cancer

In CRC regulation, the expression of all associated circRNAs was upregulated and was associated with poor prognosis. For instance, high expression of circ133 and circCSPP1 is associated with clinical tumor metastasis (112, 113), while overexpression of circMYH9, circ0030998, circEIF3K, and circSLC7A6 is associated with shorter overall survival and advanced stages (Stages III and IV) (114–117). Moreover, circ0007031 was associated with anti-5-fu chemotherapy (118), and upregulated circ0005963 enhances glycolysis and oxaliplatin resistance to increase the size of drug-resistant tumors *in vivo (* 119). In terms of biological function, circSLC7A6, circ-133, circEIF3K, circ0062682, circUBAP2, circ-ERBIN, circCSPP1, and circ0007031 promote cell proliferation and colony formation, and increase migration and invasion (112, 113, 116–118, 120–122). CircMMP1 knockdown inhibits the growth and metastasis of CRC *in vivo* and suppresses the proliferation and invasion of CRC cells *in vitro (* 123). CircMYH9 has been shown to promote the cell cycle and increase cell cycle proteins to regulate proliferation (114). Circ0030998 and circ0056618 have been shown to promote angiogenesis *in vitro* and aid in invasion and metastasis (115, 124). CircCCDC66, as a cancer promotor, is highly expressed in CRC and circCCDC66-knockdown has been shown to reduce viability, migration, and invasion, and promote apoptosis (125). Overexpression of circ0005963 has been shown to enhance glycolysis and drug resistance to increase the size of drug-resistant tumors *in vivo (* 119). Circ0136666 was reported to function as a tumor promotor by mediating Treg-mediated immune escape (126).

## Other types of tumors

In addition to the mentioned DSC with high morbidity and malignancy, there are several cases of circRNAs involved in other types of DSC, such as esophageal squamous carcinoma (ESCC), oral squamous cell carcinoma (OSCC), and tongue squamous cell carcinoma (TSCC). For instance, circOGDH is upregulated in ESCC and its overexpression is closely related to tumor size and poor prognosis. In terms of cell activities, circOGDH can promote tumor growth by promoting proliferation, metastasis, invasion, and glutamine metabolism (127). As a tumor suppressor, low expression of circ0000140 is closely associated with poor prognosis of lymph node metastasis in OSCC, and cell function testing showed that overexpression of circ0000140 blocked the proliferation, migration, and invasion of OSCC cells (128). High expression of circ0000003 in ESCC was reported to be correlated with advanced TNM stage and increased tumor size. Overexpression of circ0000003 has been shown to promote cell proliferation, whereas circ0000003 knockdown significantly inhibited cell invasion and migration (129).

## Functional mechanisms of circRNAs in the TME

The deterioration of cancers relies on the recruitment and reprogramming of tumor cells to the surrounding normal cells (130). Therefore, in the TME, the crosstalk between tumor cells, immune cells, normal cells, extracellular matrix and various signaling molecules becomes a critical factor (9, 131, 132). As mentioned above, most circRNAs have aberrant expression levels in diverse DSCs, and these dysregulations are caused by different mechanisms. In the following sections, we will focus on the functional mechanisms of circRNA in the TME (**Table 3**).

**Table 3:** Role of circRNAs in the tumor microenvironment of digestive system tumors.

Category	Target	CircRNA	Cancer type	Expression	Function to target	Refs.
Immune system	macrophages	circ0003410	HCC	up	promote polarization of M2 macrophages	(80)
	macrophages	circ110102	HCC	down	mediate chemotaxis of monocytes and TAM	(83)
	macrophages	circ00074854	HCC	up	knockdown of circ00074854 suppressed macrophage M2 Polarization	(91)
	macrophages	circTMEM181	HCC	up	interfere with the proliferation of CD8+ T cell and induce exhaustion	(89)
	NK cells	circUHRF1	HCC	up	induce natural killer cell exhaustion	(90)
	NK cells	circ0000977	PC	up	evade immune surveillance and NK cell-mediated lysis	(184)
	NK cells	circ0007456	HCC	down	increase NK cell sensitivity to tumor	(85)
	CAFs	circSLC7A6	CRC	up	promote CRC tumorigenesis	(117)
	CAFs	circUBAP2	HCC	up	promotes HCC cell migration	(87)
	CAFs	circEIF3K	CRC	up	upregulate PD-L1 and promote tumorigenesis	(116)
	Treg	circ0136666	CRC	up	Treg-mediated immune escape	(126)
ECM	MMP1	circMMP1	CRC	up	degrade fibrous collagen	(123)
	MMP1	circDLC1	HCC	down	degrade fibrous collagen	(86)
	MMP2	circ0000620	GC	up	degrade type I and IV collagen	(102)
	MMP9	circUBAP2	HCC	up	degrade type I and IV collagen	(81)
	MMP16	circ0001806	HCC	up	degrade type I fibrous collagen	(92)
	MMP19	circ0005556	GC	up	/	(109)
metabolism	glycolysis	circ0000140	OSCC	down	reduce glycolysis	(128)
	glycolysis	circ0005963	CRC	up	enhance glycolysis	(119)
	glycolysis	circZNF91	PC	up	enhance glycolysis	(98)
	glutamine metabolism	circ0000003	TSCC	up	promote glutamine catabolism, α -KG production, and ATP production	(129)
	glutamine metabolism	circOGDH	ESCC	up	elevated glutamine metabolism	(127)
	serine metabolism	circ0062682	CRC	up	promote serine metabolism	(120)
	serine metabolism	circMYH9	CRC	up	promote serine metabolism	(114)
Angiogenesis	VEGF-A	circ0030998	CRC	up	promote angiogenesis	(115)
	VEGF-A	circUBAP2	CRC	up	promote angiogenesis	(121)
	VEGF-A	circ0056618	CRC	up	promote angiogenesis	(124)
	VEGF-A	circCCT3	PC	up	promote angiogenesis	(93)
	VEGF-A	circSHKBP1	GC	up	promote angiogenesis	(99)
	VEGF-A	circRANGAP1	GC	up	promote angiogenesis	(100)
	VEGF-C	circNFIB1	PC	down	inhibit lymphangiogenesis	(96)
Hypoxia	HIF-1α	circERBIN	CRC	up	increase HIF-1α expression to promote angiogenesis	(122)
	HIF1α/VEGFA	ebv-circLMP2A	GC	up	increase HIF-1α expression to promote angiogenesis	(107)
	RhoA	circ-133	CRC	up	reduce E-cadherin	(112)
	autophagy	circCCDC66	CRC	up	promote autophagy	(125)
	PRKAA1	circC6orf132	GC	up	promotes glycolysis	(110)
EMT	COL1A1	circCSPP1	CRC	up	promote EMT	(113)
	SOX4	circ0001666	PC	up	promote EMT	(94)
	PD-L1	circ0003288	HCC	up	promote EMT	(88)
	SMAD4	circOXCT1	GC	down	promote EMT	(108)
	EGFR	circ0081143	GC	up	promote EMT	(111]
	ESRP1	circ0092367	PC	down	promote EMT	(97)
Chemotherapy resistance	drug efflux protein ABCB1	circFBXO11	HCC	up	OXA resistance	(82)
	drug efflux proteins ABCC5	circ0007031	CRC	up	5-FU resistance	(118)
	drug efflux proteins P-gp	circ0074298	PC	up	GEM resistance	(95)
	promote autophagy	circMCTP2	GC	down	CDDP resistance	(106)
	promote autophagy	circCUL2	GC	down	CIS resistance	(101)
	promote autophagy	circPVT1	GC	up	CIS resistance	(103)
	inhibit apoptosis	circARVCF	GC	up	DDP resistance	(104)
	inhibit apoptosis	circ0000260	GC	up	CDDP resistance	(105)
	inhibit apoptosis	circSORE	HCC	up	Sorafenib resistance	(84)

## CircRNAs regulate the immune system

Macrophages are released into the blood as immature monocyte precursors from bone marrow and migrate to different tissues for corresponding differentiation (133). Different forms of macrophages play different roles in the development of cancer (134). Macrophages can be classified into classical M1 macrophages and alternate M2 macrophages due to different activation procedures (135). Although tumor-associated macrophages (TAMs) are different from the M1 and M2 subtypes, they are generally similar to M2 macrophages and promote tumor growth by inducing immunosuppression (135–137). Previous studies have shown that macrophages play a dual role in cancer, promoting tumor growth and metastasis in addition to inhibiting tumor growth and escape (138–140). In most cases, TAMs promote cancer deterioration and resistance to treatment by providing nutrition and nutritional support to malignant cells (141).

Recent studies have shown that circRNAs promote or inhibit cancer progression in digestive system cancers by regulating macrophage polarization (142). For example, circ0003410, which is highly expressed in liver cancer, promotes the development of HCC by regulating the expression of macrophages. The carcinogenic effect of circ0003410 is mainly reflected in enhancing the proliferation and migration ability of liver cancer cells (80). As a highly expressed chemokine in the TME, CCL5 mainly activates and recruits M2 macrophages, induces extracellular matrix remodelling and enhances tumor cell metastasis and other procancer effects (143, 144). Additionally, the experimental results proved that high expression of circ0003410 could downregulate the anticancer effect of miR-139-3p and upregulate the expression of CCL5 to recruit M2 macrophages and increase the proportion of M2/M1 macrophages to promote cancer progression (80). The chemokine CCL2 is elevated in HCC and is connected with malignant activities and poor prognosis (145, 146), playing an essential role in promoting cancer progression by activating M2 macrophages (147). CCL2 can bind to CCR2 on the macrophage cytomembrane to increase the chemotaxis of TAMs (145). Therefore, lowering the expression level of CCL2 is likely to play a vital role in cancer suppression. Circ0110102, as a tumor suppressor, has low expression in HCC, and its downstream miR-580-5p is associated with poor outcomes, which function to upregulate the expression of CCL2 by decreasing the expression of PPARα. Thus, circ0110102 may function as a sponge of miR-580-5p to decrease the expression of CCL2 through the activation of PPARα in HCC cells (83). Additionally, some studies indicate that exosome-mediated macrophage activation plays an important role in cancer progression (148, 149). Exosomes can transmit signals between cancer cells and TAMs, thus influencing tumor progression (150, 151). Circ00074854 is another tumor promoter in HCC, and circ00074854 knockdown mediates its inhibitory effects by reducing the protein stability of an RNA-binding protein called HuR and exosomes with downregulated circ00074854, which can be delivered to macrophages to inhibit macrophage M2 polarization *in vitro (* 91).

Cancer immunotherapy was once regarded as the key node of cancer treatment; however, due to the existence of immune checkpoints such as PD1, PDL1, and CTLA-4, cancer immunotherapy is limited to a certain extent (152–154). Therefore, inhibitors that target the abovementioned immune checkpoints could change the limitations of current tumor drug resistance and have revolutionized cancer therapy (155–157). In the tumor immune microenvironment, PD1 and its ligand PDL1 can induce tumor resistance to immune-induced apoptosis, thus resisting immunotherapy and promoting cancer progression (158). PD1/PDL1 immunotherapy can block recognition between PD1 and PDL1, thus restoring normal T cell function to recognize tumor cells and prevent tumor escape (159). Overexpression of exosomal circTMEM181 derived from HCC cells targets macrophages, reshapes the immune microenvironment, and induces immunosuppression, specifically by interfering with the viability of CD8+ T cells and inducing depletion. CD39, a pivotal enzyme secreted by macrophages can activate the ATP-adenosine pathway (160, 161), and is upregulated by the circTMEM181/miR-488-3p/CD39 axis. Upregulation of CD39 can impair the signaling of extracellular ATP-stimulated immune responses in the TME, thereby impairing antitumor immunity (162, 163). For instance, CD39 and CD73 can activate the ATP-adenosine pathway through synergistic action to weaken antitumor immunity (164). The interaction between tumor cells and macrophages activates the ATP-adenosine pathway leading to hyposensitivity to anti-PD1 therapy (165, 166). Therefore, abnormal CD39 expression targeting macrophages is expected to be developed as a treatment strategy to reverse resistance to PD1. Additionally, the depletion of CD39 or macrophages has been verified *in vivo* to inhibit HCC progression and promote CD8+ T cell exhaustion (89, 144). In addition to macrophages, NK cells are involved in PD1-mediated tumor immunity and drug resistance (167). Extracellular circUHRF1 produced by HCC cells may be a key factor in reducing the immune evasion by impairing NK cell-associated functions. CircUHRF1 suppresses the activity of miR-449c-5p to upregulate TIM-3 expression, which functions as an inhibitory receptor on NK cells to reduce antitumoral immunity (168, 169). Therefore, reducing circUHRF1 expression may be developed as a novel approach to recover sensitivity to anti-PD1 therapy (90).

Cancer immunosurveillance is an important process for the immune system to monitor, recognize, and destroy tumor cells, including elimination, balance, and escape (170–172). In the first stage, innate immune cells kill cancer cells through tumor cell recognition and proinflammatory cytokines. In the second stage, the cancer cells develop resistant clones that cannot be eliminated; at this point, if the cancer cells cannot be eliminated by other means, they will enter the final stage - escape (173, 174). As a highly immunosuppressive subgroup of CD4+ T cells, Tregs regulate the expression of the transcription factor FoxP3 (175), which is positively related to poor outcomes in various cancers by downregulating antitumor immune responses (176).

In CRC, upregulation of circ0136666 promotes PD-L1 and Treg activation by sponging miR-497. Studies have shown that silencing circ0136666 reduces CD4^+^ and FOXP3^+^, and upregulates CD8^+^ Tregs, thereby facilitating immune escape mediated by Tregs through the miR-497/PD-L1 pathway (126). In addition to the abovementioned Tregs, NK cells also play an essential role in immune escape. As the major effector in congenital immunity, NK cells can kill tumor cells in the initial stage (177) and suppress tumor metastasis. When NK cells are inhibited by tumor-derived molecules or related factors, their ability to target tumor cells quickly and effectively will be impaired, ultimately abolishing the process of cancer progression and immune escape (178, 179). circ0007456 acts as a sponge of miR-6852-3p, which can affect ICAM-1, a cell surface glycoprotein and an adhesion receptor that can regulate tumor development and metastasis (180). ICAM-1 can increase the effect of NK cells on tumor cells, while its downregulation is a key mechanism by which cancer cells evade NK cell attack (181). circ0007456 upregulates ICAM-1 by sponging miR-6852-3p in HCC, and overexpression of ICAM-1 has been shown to increase the sensitivity of tumor cells to NK cells (85). Thus, circ0007456 may represent a promising biomarker of HCC, and targets for immune avoidance. In the TME of pancreatic cancer, hypoxia induces the overexpression of circ0000977, increases the expression of HIF1A and ADAM10, enables tumor cells to avoid immune surveillance and suppresses the lethal effect of NK cells on pancreatic cancer cells (182, 183). However, studies have shown that knocking down circ0000977 attenuates the inhibition of miR-153, whereas high expression of miR-153 attenuates the lethal effect and reduces HIF1α-mediated immune escape (184). The circ0000977/miR-153/HIF1A/ADAM10 axis may be used as an immune sensitizer to treat and/or prevent cancer. Therefore, circRNA-mediated tumor immune surveillance and immune escape in the TME may be vital breakthrough directions for future immunotherapy research.

## Cancer-associated fibroblasts and cancer deterioration

CAFs are multifunctional fibroblasts in the TME, whose functions include matrix remodelling, signal transduction with cancer cells, and crosstalk with infiltrating leukocytes and diverse chemokines (185–188). CAFs regulate cancer metastasis and influence angiogenesis and therapeutic response through synthesis and remodelling of the extracellular matrix (ECM) and production of growth factors (189–192). Therefore, if the above cancer-promoting characteristics of CAFs can be targeted for treatment, it will optimize current cancer treatment strategies.

Chemokines have become important participants in the tumorigenesis process (193). CXCL13 and its homologous receptor CXCR5 have demonstrated outstanding abilities to regulate tumor growth, and play crucial roles in inflammation, cancer, and immune responses (194, 195). Matrine, as an alkaloid extracted from traditional Chinese medicine, has anticancer effects (196, 197). In CRC, it inhibits cancer development by inhibiting the secretion of the exosomal circSLC7A6 from CAFs (117). *In vitro* experiments showed that CXCR5 overexpression reversed the inhibitory effect of matrine on invasion and apoptosis to promote cancer progression. The interaction of exosomal circSLC7A6 with the miR-21, miR-107, and miR-200 families may be a miRNA-dependent means to regulate CXCR5 (198, 199). CXCL11 is another CAF-derived chemokine ligand that participates in the progression of various cancers and mediates the recruitment of T cells, NK cells, and macrophages, predominantly through the receptor CXCR3 (200, 201). The mRNA expression level of CXCL11 is highly expressed in HCC tissues, especially in metastatic HCC compared to the nonmetastatic. Moreover, CXCL11 endows HCC cells with stronger metastasis and cell proliferation ability (202). Bioinformatic analysis and cell experiments confirmed that tumor metastasis regulated by CXCL11 is mediated by circUBAP2. Additionally, circUBAP2 participates in the functions of CXCL11 derived from CAFs *via* the circUBAP2/miR-4756/IFIT1/IFIT3 axis (87). Hypoxia in the TME is also an important factor in stimulating circRNA secretion by CAFs (203). By culturing CAFs in a hypoxic environment, Yang et al. found that CAFs can secrete exosomes, some of which can secrete circEIF3K. circEIF3K has been shown to promote CRC progression in patients and exerted an oncogenic role in animal and clinical studies by downregulating its downstream effector miR-214 to attenuate the invasion of hypoxia-mediated CRC cells and downregulate PD-L1 expression. Therefore, circEIF3K secreted by CAFs stimulated by hypoxia blocks the expression of its anticancer effects *via* the miR-214/PD-L1 axis (116). Since circRNA can regulate tumor metastasis by regulating CAFs, it may open up a new strategy for targeted therapy against CAFs.

## CircRNAs regulate energy metabolism in the TME

Cancer cells undergo metabolic reprogramming to maintain bioenergetics, redox states, cell signaling, and biosynthesis, which are often poorly vascularized, nutrition-deficient microenvironments (204). Recently, energy metabolism has been regarded as a new hallmark of cancer, which has shifted the research focus toward tumor metabolism (205, 206). In the TME, the crosstalk between complex substance metabolism and circRNAs greatly affects host metabolism and dynamic balance, thus affecting cancer progression. Some circRNAs can influence these behaviors by regulating metabolism (207).

Glucose is the main energy source for almost all cells, including cancer cells, and increased glucose uptake is associated with the proliferation and metastasis of cancer cells (208). Although cancer cells consume a large amount of glucose, they have a strong ability to convert it into lactic acid due to disproportionate oxygen intake (209), and hypoxic tumor glycolysis is more likely to promote metastasis (210). Circ0000140 has been shown to have low expression in OSCC, and overexpressed circ0000140 has been shown to inhibit glycolysis in OSCC cell lines by significantly inhibiting GLUT1 and LDHA protein levels. The above inhibitory effect was caused by sponging miR-182-5p and upregulating CDC73 (128). Circ0000140 has been shown to hinder glycolysis metabolism *via* the miR-182-5p/CDC73 axis to affect proliferation, migration, and invasion. In addition to influencing tumor invasion and metastasis by regulating glucose metabolism in the TME, circRNA plays a significant role in chemical resistance (211). Malignant tumors often produce ATP rapidly through glycolysis, which facilitates the rapid proliferation of tumor cells and the generation of chemotherapy resistance (212). The M2 isoform of pyruvate kinase (PKM2) plays a significant role in catalyzing glycolysis (213). Circ0005963 has been proven to function as a sponge for miR-122 to target PKM2. A previous study demonstrated that overexpression of circ0005963 and PKM2 protein in sensitive cells played a vital role in accelerating glycolysis and promoting drug resistance. Additionally, the inhibition of circ0005963 attenuated glycolysis and reversed chemoresistance to oxaliplatin *via* the circ0005963/miR-122/PKM2 axis (119). The high expression level of circZNF91 secreted by pancreatic cancer cell exosomes in a hypoxic environment significantly promoted chemical resistance. Mechanistically, circZNF91 binds to miR-23b-3p and inhibits the suppression of miR-23b-3p on SIRT1. Moreover, overexpression of SIRT1 leads to the increased glycolysis and GEM chemoresistance by enhancing the stability of HIF-1α (98). Additionally, high expression of circC6orf132 in hypoxic GC cells promotes cell proliferation and glycolysis through the miR-873-5P/PRKAA1 axis (110).

Amino acid metabolism is involved in the development of tumors, and the maladjustment of glutamate metabolism not only promotes the growth of tumor cells but also promotes tumor invasion and metastasis (204, 214). Glutamine is a major nutrient in plasma, and glutaminase can convert glutamine into α-KG to participate in the TCA cycle, carbon and nitrogen metabolism in cells, and provide energy for cells (215–218). Additionally, other studies have shown that glutamine metabolism promotes the malignant proliferation of cancer cells by participating in macromolecular biosynthesis and regulating REDOX homeostasis and signaling pathways (219). In TSCC, circ0000003 upregulates the expression of GLS to promote glutaminase expression and cell proliferation by sponging miR−330−3p. Meanwhile, the miR−330−3p inhibitor has been shown to reverse the above downgrading effects (129). Similarly, circ-OGDH has been shown to elevate glutamine metabolism by sponging miR-615-5p to release PDX1. Further studies showed that increased miR-615-5p decreased glutamine consumption, α-KG production, and ATP content, all of which were inhibited by PDX1 (127).

Beyond that, the serine biosynthesis pathway represents the key point in glucose conversion (220). Serine from synthetic and exogenously ingested glycolysis branches can be converted to glycine and provides a one-carbon unit for one-carbon metabolism (221). The one-carbon unit can regulate the proliferation of cancer cells by affecting nucleotide synthesis, methylation pathways, and redox balance (222, 223). Circ0062682 exerts its carcinomatous functions by sponging miR-940, which targets PHGDH (120). As the first step rate-limiting enzyme in the serine biosynthetic pathway, PHGDH promotes cancer cell proliferation through overexpression in various cancers (224). Studies proved that circ0062682 knockdown could decrease the expression of serine and glycine, scale down NADPH/NADP+ and GSH levels by downregulating PHGDH, thus inhibiting the proliferation of CRC cells. Further research showed that cancer cells can adapt to nutrient stress conditions by increasing serine biosynthesis. The expression of circ0062682 and PHGDH was increased under serum starvation. Serine deprivation could induce the expression of circ0062682, then upregulated PHGDH expression *via* the miR-940/PHGDH axis (120). In the same way, circMYH9 promoted serine and glycine metabolism through p53−mediated upregulation of PHGDH (114, 225). The results proved that circMYH9 could regulate REDOX homeostasis by inhibiting p53 *via* m6A modification and upregulating the downstream target PHGDH (114). As a feature of rapid proliferation of tumor cells, circRNAs can meet the high metabolic demand of tumor cells by regulating glycolysis, serine metabolism and glutamine metabolism. Further research in this field may help us to obtain the key to decipher the malignant proliferation of tumor cells.

## Angiogenesis

Tumor cells have very high nutrient requirements to maintain their anabolic demand and energy production efficiency. Tumor angiogenesis can meet the current stringent energy requirements by increasing the uptake of extracellular nutrients (207). Due to the lack of vasculature, tumor growth is limited when the tumor size exceeds 2–3 mm. Here, the angiogenesis switch is triggered to enable survival and promote the invasion and metastasis of cancer cells (226). Angiogenic factors and cytokines in the TME promote tumor angiogenesis (227). In the TME, circRNA plays a critical role in proangiogenic and antiangiogenic signaling networks.

VEGFA is a major factor driving tumor vascular bed dilation. As a part of the growth factor family, VEGFA has a strong ability to promote the angiogenic environment, such as by increasing microvascular density and vascular permeability, finally, leading to tumor resistance to angiogenic therapy (228, 229). Overexpression of circ0030998 can promote angiogenesis by sponging miR-567 to increase VEGFA expression. High expression of VEGFA further promotes cell cycle progression and HUVEC tubular structure formation (115). The latest research shows that circUBAP2 promotes CRC cell progression and angiogenesis *via* the miR-199a/VEGFA axis. miR-199a directly interacts with VEGFA and significantly suppresses its expression level, which can be reversed by upregulation of circUBAP2 (121). Circ0056618 has been shown to promote angiogenesis by sponging miR-206 and upregulating CXCR4 and VEGFA (124). CircCCT3 functions as a miR-613 sponge to upregulate the expression of VEGFA and VEGFR2 to promote angiogenesis (93). Additionally, knockdown of circCCT3 reversed the above carcinogenic effects. CircSHKBP1 is an upregulated circRNA in GC tissue that can be efficiently delivered into the circulation by exosomes. CircSHKBP1 sponges miR-582-3p and increases HUR levels (99), to prevent rapid mRNA degradation and promote high and stable VEGFA expression by stabilizing VEGFA mRNA expression (230). Additionally, circSHKBP1 directly binds to HSP90 and inhibits the ubiquitination of STUB1 to HSP90. Tumorigenesis of circSHKBP1 can be blocked by anti-VEGF antibodies and HSP90 inhibitors (99). Wang et al. reported that circ-RANGAP1 acted as a sponge for miR-877-30 to promote GC angiogenesis through the miR-877-3p/VEGFA axis (100).

In addition to spreading directly through the blood to other tissues and organs, cancer cells can also spread through the lymphatic system (231). Tumors trigger lymphatic growth and remodelling by secreting lymphangiogenic growth factors such as VEGF-C (232, 233). VEGF-C can bind with VEGFR3 and activate related signaling pathways, promoting lymphatic endothelial cell proliferation and vascular permeability (234). CircNFIB1 is crucial for inhibiting LN metastasis in PDAC. Mechanistically, miR-486-5p promotes lymphangiogenesis in PDAC cells, whereas circNFIB1 directly binds to miR-486-5p to inhibit its expression. PIK3R1, a regulatory subunit of PI3K, suppresses the activation of the PI3K/Akt pathway and inhibits cancer progression (235, 236). miR-486-5p degrades PIK3R1 by targeting its 3’UTR, whereas circNFIB1 reverses the negative regulation of miR-486-5P on PIK3R1 expression by inhibiting the phosphorylation of Akt and increasing the expression of PI3K3R1 (96). As another member of the VEGF family, VEGF-D regulation by circRNA has not yet been reported in DSCs, but its role in promoting lymphatic metastasis in bladder cancer has been confirmed (22). Therefore, the mechanism by which circRNA regulates VEGF-D in DSCs needs to be explored and verified.

In conclusion, circRNAs promote angiogenesis primarily by mediating the VEGFA family, creating a favorable microenvironment for nutrient requirements and metastasis of tumor cells. Therefore, targets against circRNAs have great potential as future inhibitors of angiogenesis.

## Hypoxia regulates circRNA production in the TME

Rapid and unrestricted proliferation limits oxygen and blood supplementation and induces hypoxia, which is a representative microenvironmental feature of almost all solid tumors (237). Rapid tumor proliferation can also stimulate the generation of new vascular systems. In this case, the vasculature becomes disordered and the distance between capillaries increases beyond the ability of oxygen diffusion, which is not conducive to the transport of oxygenated blood (238). As tumor cells adapt to hypoxia, they result in a more malignant and therapeutic resistant tumor phenotype (239).

During this process, the overexpression of HIF-1α is a significant marker. HIF-1α enhances the activity of Snail and Twist and then reduces E-cadherin expression, thereby promoting invasion, a cancer stem cell-like phenotype, and chemoresistance (240). Recently, circRNA has also been shown to be regulated by hypoxia in the TME (112, 122). Hypoxia and TGF-β stimulate the expression of circERBIN, which promotes HIF-1α through a protein called 4EBP-1, which plays a vital role in HIF1-α translation (241). Further studies have shown that 4EBP-1 is a common downstream target for both miR-125a-5p and miR-138-5p, with a negative and positive correlation with circERBIN, respectively. These results suggest that circERBIN increases HIF-1α expression *via* the miR-125A-5p/miR-138-5p/4EBP-1 signaling pathway (122). HIF1α has been shown to upregulate ebv-circLMP2A under hypoxia to enhance angiogenesis, while ebv-circLMP2A stabilizes HIF1α by reducing VHL (242). Mechanistically, ebv-circLMP2A interacts with KHSRP to enhance the degradation of VHL mRNA, thereby activating the HIF1α/VEGFA axis to promote angiogenesis. HIF1α and ebv-circLMP2A under hypoxia promote the increased expression of each in the form of positive feedback (107). In the case of hypoxia, adaptive reprogramming is mediated by the HIF-1 protein in most cases. Hypoxia contributes to the upregulation of circCCDC66 in CRC. Upregulated circCCDC66 can increase the viability of CRC cells in the hypoxic environment, enhance invasion and migration ability, and inhibit apoptosis. While circCCDC66 knockdown can completely reverse its carcinogenic effect by decreasing the inhibition of miR-3140, the increased miR-3140 resulted in the expression of autophagy regulator Beclin1 to inhibit cancer progression by inhibiting autophagy (125). The interior of solid tumors can be roughly divided into hypoxic and normoxic cancer cells (243). After incubation with hypoxic-derived exosomes, prometastatic signals can be transmitted to normoxic cancer cells, resulting in overexpressed GEF-H1/RhoA and increased cell migration capacity. GEF-H1 and RhoA can be directly targeted by miR-133a and negatively regulated. Circ-133 can be delivered into relatively normoxic cells and targeted to GEF-H1/RhoA by sponging miR-133a, which serves to decrease the expression of E-cadherin on the surface to enhance the migration capacity of cancer cells (112). Therefore, hypoxia can regulate the expression of circRNAs, and thereby indirectly promote angiogenesis, invasion and metastasis in which circRNAs are involved.

## CircRNA induces EMT and tumor cell migration

EMT is a cellular phenomenon that allows stationary polarized epithelial cells to undergo various morphological changes resulting in a migratory, invasive mesenchymal phenotype (244–246). Activation of EMT under pathological conditions has a significant role in the initiation of tumor development and metastasis and is involved in the transformation of epithelial cells into mesenchymal cells, leading to tumor cell migration (247–249).

Wang et al. demonstrated that circCSPP1 was upregulated in CRC and that circCSPP1 sponges miR-193a-5p to mitigate its inhibition of COL1A1, thereby promoting the progression of EMT. In contrast, knockdown of circCSPP1 decreased the expression of N-cadherin and vimentin by downregulating COL1A1 and increased the expression of E-cadherin, weakening the aggressiveness of tumor cells (113). Similarly, Zhang et al. demonstrated that overexpression of circ0001666 leads to low expression of E-cadherin and upregulation of Vimentin, and promotes EMT in pancreatic cancer cells. Mechanistically, circ0001666 acts as a sponge for miR-1251 to weaken the inhibition of miR-1251 on the downstream target SOX4, and the high expression of SOX4 in turn regulates the EMT of cancer cells by upregulating the expression of EZH2 and promoting the invasive properties of pancreatic cancer cells (94). Xu et al. reported that HepG2 and Huh7 cells overexpressing miR-145 had increased expression of E-cadherin and reduced expression of N-cadherin, leading to reduced migration and invasion abilities. These findings suggest that miR-145 can inhibit the EMT of HCC cells. Circ0003288 can reduce the inhibitory effect of miR-145 on PD-L1 expression and EMT by acting as a miR-145 sponge (88). In addition, circ-OXCT1 forms a spongiform structure with miR-136, thereby inhibiting SMAD4 expression and EMT through the circOXCT1/miR-136/SMAD4 axis in GC cells (108). Knockdown of circ0081143 alleviates hypoxia-induced migration and EMT *via* the miR-497-5p/EGFR axis (111). Overexpression of circ0092367 functioned as a tumor suppressor to induce ESRP1 expression by acting as a sponge for miR-1206 to reduce its expression, thereby inhibiting pancreatic cancer cell invasion and EMT (97). EMT plays an important role in the malignant metastasis of tumor cells. However, the research on circRNAs in EMT is still limited, so the specific process of circRNAs regulating EMT still needs further research.

## CircRNAs regulate the ECM in the TME

The TME mainly consists of stromal cells and ECM components. The ECM is secreted by cells to provide structural and biochemical support and has a significant role in cell proliferation, differentiation, and maintenance of tissue homeostasis (250–252). Collagen and fibronectin, as the most important components of ECM proteins, are regulated by the MMP family of proteins (253, 254). Recent studies have shown that circRNA abnormally regulates the MMP family to cause remodelling of the ECM matrix, thus supporting tumor cell invasion into the basement membrane and stroma, vascular infiltration and metastasis (255–257).

CircMMP1 is highly expressed in CRC and can enhance MMP1, MMP2, and MMP9 by sponging miR-1238 to promote remodelling of the ECM matrix and metastasis of cancer cells (123). CircDLC1 is a tumor suppressor with low expression in HCC, and the overexpression of MMP1, MMP2, MMP3, and MMP10 remarkably appeared in circDLC1-knockdown cells. Mechanistically, circDLC1 impaired mRNA stability and translation by competitively binding to the mRNA stabilizing protein HuR, thus downregulating the expression level of MMP1. In conclusion, these data suggest that circDLC1 can inhibit HCC metastasis through the HUR-MMP1 axis (86). Circ0000620 is upregulated as a carcinogen in GC tissues, while circ0000620 acts as a sponge for miR-671-5p to upregulate MMP2 expression, thereby promoting metastasis and angiogenesis (102). MMP9 acts primarily as collagenase and mainly degrades type IV collagen (258, 259). CircUBAP2 is upregulated in HCC and upregulates MMP9 by sponging miR-194-3p to promote HCC metastasis (81). Circ0001806 adsorbs miR-193a-5p and negatively modulates its expression to upregulate the expression of MMP16 to degrade type I fibrous collagen (260), which could enhance the proliferation, migration, and invasion of HCC cells (92). Additionally, in GC tissues, overexpression of circ0005556 can accelerate GC progression by increasing MMP19 expression by sponging miR-4270 (109). However, current studies on MMP19 only focus on its functional phenotype, and there are few specific mechanisms for MMP19 to regulate ECM. ECM remodeling occurs in the whole process of tumorigenesis and development, and plays a vital role in supporting tumor cells to invade basement membrane, stroma and vascular penetration. Therefore, the regulation of ECM by circRNA can be used to create the targeted treatment for high tumor aggressiveness.

## CircRNAs regulate chemotherapeutic resistance

**Figure 4:**
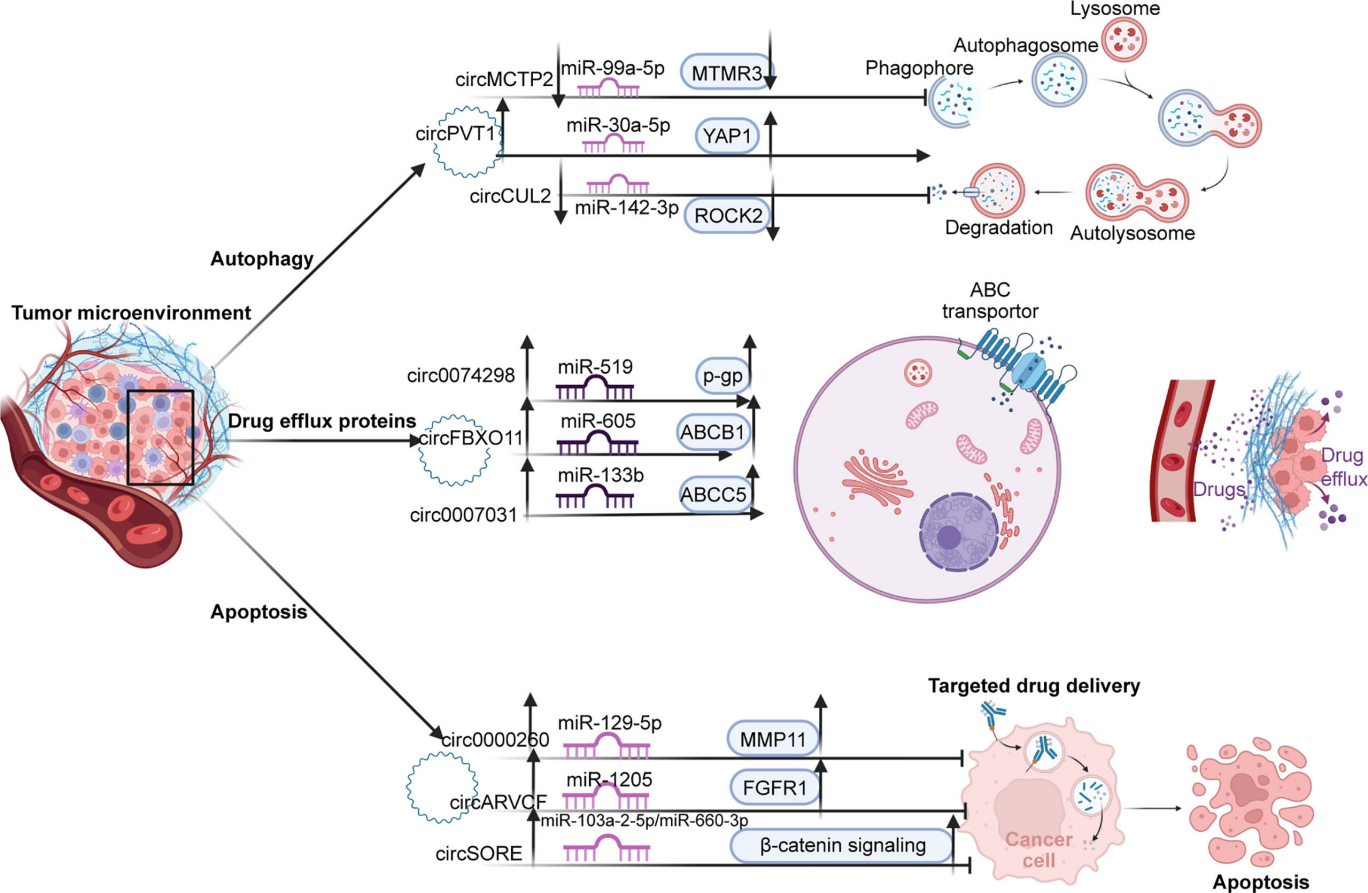
There are three main pathways of resistance to TME. First, by promoting autophagy; upregulated circPVT1 promotes autophagosome formation through the mir-99a-5p/MTMR3 axis. In contrast, cirMCTP2 and cricCUL2, as tumor suppressors, inhibit autophagy through downregulated MTMR3 and ROCK2 expression, respectively. Second, by regulating the expression of drug excretion proteins, circ0074298, circFBXC11, and circ0007031 promote the excretion of chemotherapy drugs from cells by promoting the expression of p-GP, ABCB1, and ABCC5 proteins, respectively. Thirdly, by inhibiting apoptosis. Circ0000260, circARVCF, and circSORE inhibit drug-induced apoptosis by activating the MMP11, FGFR1, and β-catenin pathways, resulting in drug resistance.

Although chemotherapy remains the preferred method of postoperative cancer treatment, a significant proportion of cancer patients still experience local recurrence and distant metastasis due to the development of drug resistance. Chemotherapeutic resistance is an obstacle to patients’ long-term survival (261–263). Cancers use different pathways to evade treatment-induced cell killing and acquire drug resistance in the TME, which is a huge barrier to cancer treatment (264, 265). In recent years, many studies have shown that circRNAs are important players in regulating drug resistance through various mechanisms such as drug efflux proteins, autophagy, and apoptosis (266). Therefore, understanding the regulatory mechanisms of circRNA associated with chemotherapeutic resistance can identify new targets to optimize treatment. (**Figure 4**)

Among the various drug resistance mechanisms, drug efflux is one of the first methods used in cancer cells. Cancer cells preferentially use ATP-fuel ATP binding box (ABC) transporters to squeeze out chemotherapeutic drugs and block their killing effects by efflux of drugs, leading to the development of drug-resistant phenotypes (267). There is considerable evidence that the expression of the ATP binding box (ABC) transporter, particularly by member 1 of the ABC subfamily B (ABCB1), confers resistance to cytotoxicity and is targeted chemotherapy (268). Qin et al. demonstrated that circFBXO11 promoted tumor progression and OXA resistance in HCC cells. Overexpression of circFBXO11 can upregulate downstream target FOXO3 through sponging miR-605, and overexpression of transcription factor FOXO3 can promote the level of ABCB1 protein in HCC cells, thereby promoting OXA efflux of HCC cells to produce drug resistance (82). It has also been reported that circ0007031 sponges miR-133b to regulate the expression of the downstream target ABCC5. Experiments have proved that circ0007031, which is highly expressed in CRC, promotes the efflux of 5-FU in cancer cells by upregulating ABCC5 and increases the drug resistance of CRC to 5-FU (118). Chen et al. demonstrated that highly expressed circ0074298 regulates the expression of SMOC2 by sponging miR-519d and affects biological behavior such as resistance to gemcitabine in pancreatic cancer. Additionally, knockdown of circ0074298 increased the sensitivity of PANC-1-GEM cells to gemcitabine and downregulated the expression levels of MDR1 and SMOC2 (95). However, the mechanism by which SMOC2 exerts drug resistance in pancreatic cancer remains to be elucidated.

Autophagy is a conserved self-degrading system and a tightly coordinated process. On the one hand, autophagy can suppress tumor progression by removing misfolded proteins and dysfunctional organelles (269–271) On the other hand, autophagy is essential for maintaining cellular homeostasis under stress, and in the later stages of tumor growth, autophagy can be used to survive in the absence of nutrients or oxygen (272, 273). In digestive cancers, autophagy is a significant mechanism of cell survival that is effectively used by tumor cells. Through cell degradation, the recovery of intracellular substrates and damaged organelles can alleviate cellular stress caused by nutrient deprivation, hypoxia, radiation, and cytotoxic agents (240, 274, 275). Recent studies have shown that circRNAs can promote drug tolerance induced by autophagy by regulating related miRNAs and downstream targets (276). For instance, circMCTP2 has been shown to be downregulated in CDDP-resistant GC cells. Mechanistically, circMCTP2 acts as a sponge for miR-99a-5p and promotes the expression of the downstream target MTMR3. Upregulated circMCTP2 and MTMR3 have been shown to inhibit autophagy in CDDP-resistant GC cells and induce apoptosis in response to CDDP treatment (106). Circ-PVT1 promotes cancer cell resistance through the miR-30A-5p/YAP1 axis in cisplatin chemotherapy for GC. Circ-PVT1 knockdown repressed DDP resistance by inducing apoptosis and inhibiting autophagy. Overexpression of miR-30a-5p could reduce DDP resistance by suppressing YAP1 (103). Lei et al. reported low expression of circCUL2 as a tumor suppressor in GC tissues. Mechanistically, circCUL2 inhibits autophagy in cisplatin-resistant GC cells through the miR-142-3p/ROCK2 axis (101).

Most cancer treatments eliminate tumor cells by triggering apoptosis, but there are also corresponding antiapoptotic signals in cancer, particularly the activation of antiapoptotic mechanisms, which enable tumor cells to escape apoptosis and conduct uncontrolled malignant proliferation (277). Recently, reports on circRNAs have suggested that circRNAs can mediate the proapoptotic effect of chemotherapy drugs by influencing the expression of apoptosis factors and apoptosis-related signaling pathways through the signaling network (278). CircARVCF has been reported to be upregulated and increase FGFR1 expression by sponging miR-1205 in DDP-resistant GC tissues and cells. CircARVCF inhibition has been shown to decrease the expression of MRP1, MDR1, and Bcl-2, and increase the expression of Bax. Meanwhile, miR-1205 overexpression inhibited DDP resistance by downregulating FGFR1 expression (104). Circ0000260 has been shown to be highly expressed in CDDP-resistant GC cells, and knockdown of circ0000260 enhanced the chemical sensitivity of CDDP and then blocked malignant behavior. Upregulated circ0000260 downregulates CDDP chemical sensitivity *in vivo* by targeting the miR-129-5p/MMP11 axis (105). Furthermore, MMP-11 promotes tumor development in the early stage by inhibiting apoptosis and promoting the migration and invasion of cancer cells (279). Sorafenib-resistant HCC cells express high levels of circRNA-SORE, and apoptosis of sorafenib-resistant cells has been shown to increase after circRNA-SORE knockout. circRNA-SORE maintains sorafenib resistance by acting as a miRNA sponge for miR-103a-2-5p and miR-660-3p. More importantly, miR-103a-2-5p or miR-660-3p alone or in combination can significantly suppress the Wnt2b and β-catenin downstream signals (84). Activation of β-catenin to enhance cancer stem cell characterization and drive sorafenib resistance in HCC has also been reported in other articles (280).

Most of the chemotherapeutics currently in clinical use the apoptotic signaling pathway to induce cancer cell apoptosis. Thus, defects in apoptotic pathways may lead to tolerance, which limits the efficacy of treatment. Therefore, better insight into the regulation of circRNAs on the apoptotic signaling pathway may improve the curative effect of chemotherapeutics.

## Significance of exosomal circRNA for digestive system tumors

Exosomes were initially thought to be extra waste products produced by cells, and with further research, exosomes have been found to exist in various body fluids and to mediate communications between diverse cells (281). CircRNAs can also play an important role in the TME in the form of exosomes, such as participating in tumor angiogenesis, invasion, metastasis and EMT (247).

In digestive system cancers, circRNAs with carcinogenic effects are usually expressed stably in cancer cells, but they can also be encapsulated by tiny vesicles in exosomes to deliver some vital biological information from the interior of tumor cells and affect the expression of downstream miRNAs, thus affecting the progression of related cancers (282, 283). For example, circ-100338 derived from HCC cell lines can be transferred to adjacent cells by exosomes and promote the proliferation and angiogenesis of surrounding HUVECs, as well as the invasion and metastasis of HCC cells (284). Exosomes secreted by TAM contained highly expressed hsa_circ_0020256, which enters cholangiocarcinoma (CCA) cells and causes upregulation of hsa_circ_0020256, and further upregulates E2F3 by sponging miR-432-5p, thus enhancing the proliferation, invasion and metastasis of CCA cells (285). In addition to promoting cancer progression, exosome circRNA also acts as a tumor suppressor. As a tumor suppressor, when GC cells continuously ingested exosomal circRELL1, the ectopic high expression of circRELL1 can greatly weaken the proliferation and metastasis ability of GC cells. Mechanistically, circRELL1 upregulates EPHB3 by acting as a miRNA sponge for miR-637, thereby reducing inhibition of tumor growth and metastasis *in vivo (* 286). In HCC patients, circ-0051443 is delivered from normal cells to HCC cells by exosomes and inhibits malignant biological behavior by promoting apoptosis and blocking the cell cycle. The above tumor suppressive effects include reducing the weight and volume of xenograft tumors *in vivo*, mainly through circ-0051443 sponging miR-331-3p and indirectly increasing the expression of downstream target BAK1 (287).

Exosomal circRNA is more abundant than linear RNA in serum exosomes and is more easily detected with higher specificity, Therefore, exosomal circRNA has more potential as a cancer marker (288, 289). For example, circLPAR1 is stably encapsulated in exosomes, and with the progression of CRC, circLPAR1 with inversely low expression can be significantly detected in exosomes in blood, and the level of circLPAR1 in exosomes increases significantly after colorectal cancer resection (290). Hsa_circ_0015286 was significantly up-regulated in plasma exosomes and cancer cells of GC patients, and hsa_circ_0015286 was significantly down-regulated in plasma exosomes 10 days after surgery (291). In addition, highly expressed exosomal circAKT3 was associated with a higher risk of HCC recurrence and mortality (292). Together, exosomal circRNA can be used as a valuable biomarker to provide some novel insights into the diagnosis, prognosis and treatment of digestive cancer.

## Conclusion

In this review, we explored the role of circRNAs in regulating cancer progression in the digestive system tumor TME and the great potential of circRNAs as therapeutic targets and prognostic indicators. In summary, although circRNAs are in the initial stage of the TME, they have shown excellent development prospects. Therefore, it is necessary and urgent to translate circRNA research into clinical application.

## Author contributions

WG, SZ, and YH designed and guided the study. ZW, XY, and YH wrote and edited the manuscript. XY helped with reference collection. All authors read and approved the final manuscript.

## Funding

This work was supported by the National Natural Science Foundation of China (81902832), Leading Talents of Zhongyuan Science and Technology Innovation (214200510027), Henan Provincial Medical Science and Technology Research Plan (SBGJ202102117 and SBGJ2018002), Henan Medical Science and Technology Joint Building Program (LHGJ20210324), Science and Technology Innovation Talents in Henan Universities (19HASTIT003), Outstanding Foreign Scientist Studio in Henan Province (GZS2020004), and the Gandan Xiangzhao Research Fund (GDXZ2022002).

## Acknowledgments

All five figures in this manuscript were created with BioRender.com.

## Conflict of interest

The authors declare that the research was conducted in the absence of any commercial or financial relationships that could be construed as a potential conflict of interest.

## Publisher’s note

All claims expressed in this article are solely those of the authors and do not necessarily represent those of their affiliated organizations, or those of the publisher, the editors and the reviewers. Any product that may be evaluated in this article, or claim that may be made by its manufacturer, is not guaranteed or endorsed by the publisher.

## Abbreviations

circRNAs, Circular RNAs; ncRNAs, non-coding RNAs; DSC, Cancer of the digestive system; TME, tumor microenvironment; pre-mRNAs, precursor mRNAs; Pol II, RNA polymerase II; miRNAs, MicroRNAs; HuR, human antigen R; m6A, N6-methyladenosine; NK cells, natural killer cells; HCC, Hepatocellular carcinoma; PC, Pancreatic cancer; GC, Gastric cancer; CRC, Colorectal cancer; ESCC, esophageal squamous carcinoma; OSCC, oral squamous cell carcinoma; TSCC, tongue squamous cell carcinoma; LN, lymph node; EMT, epithelial-mesenchymal transformation; PDAC, pancreatic ductal adenocarcinoma; CIS, cisplatin; CDDP, cisplatin; EBv, Epstein-Barr virus; 5-fu, 5-fluorouracil; Treg, Regulatory T cells; TAM, tumor-associated macrophages; CCL2, C-C motif chemokine ligand 2; CCL5, C-C chemokine ligand 5; CCR2, C-C chemokine receptor type 2; PPARα, Peroxisome proliferator-activated receptor α; PD1, programmed cell death protein 1; PD-L1, Programmed cell death ligand-1; CTLA-4, Cytotoxic T-lymphocyte-associated protein 4; ICAM-1, Intercellular adhesion molecule 1; HIF-1α, Hypoxia-inducible factor-1α; CAFs, Cancer-associated fibroblasts; ECM, extracellular matrix; CXCL13, chemokine ligand 13; CXCR5, chemokine receptor 5; REDOX, reduction/oxidation; GLUT1, glucose transporter type 1; LDHA, lactate dehydrogenase A; PKM2, M2 isoform of pyruvate kinase; TCA cycle, the tricarboxylic acid cycle; α-KG, α-ketoglutarate; PHGDH, phosphoglycerate dehydrogenase; GSH, Glutathione; VEGFA, vascular endothelial growth factor A; HUVEC, human umbilical vein endothelial cells; VEGFR, vascular endothelial growth factor receptor; miRSC, miRNA induced silencing complex; TGF-β, transforming growth factor-β; 4EBP-1, eukaryotic translation initiation factor 4E binding protein 1; SOX4, SRY-related high-mobility-group box 4; EGFR, Epidermal Growth Factor Receptor; ESRP1, epithelial splicing regulatory protein 1; MMP, Matrix metalloproteinases; ABC, ATP-binding cassette; OXA, Oxaliplatin; MDR1, Multidrug Resistance Protein 1; SMOC2, SPARC-related modular calcium-binding protein 2; MTMR3, myotubularin-related protein 3; YAP-1, Yes-associated protein 1; FGFR1, fibroblast growth factor receptor; MRP1, multidrug resistance-associated protein 1; Bax, Bcl-2-associated X protein; BCL-2, B cell lymphoma-2

## Glossary

**Table:**

circRNAs	Circular RNAs
ncRNAs	non-coding RNAs
DSC	Cancer of the digestive system
TME	tumor microenvironment
pre-mRNAs	precursor mRNAs
Pol II	RNA polymerase II
miRNAs	MicroRNAs
HuR	human antigen R
m6A	N6-methyladenosine
NK cells	natural killer cells
HCC	Hepatocellular carcinoma
PC	Pancreatic cancer
GC	Gastric cancer
CRC	Colorectal cancer
ESCC	esophageal squamous carcinoma
OSCC	oral squamous cell carcinoma
TSCC	tongue squamous cell carcinoma
LN	lymph node
EMT	epithelial-mesenchymal transformation
PDAC	pancreatic ductal adenocarcinoma
CIS	cisplatin
CDDP	cisplatin
EBv	Epstein-Barr virus
5-fu	5-fluorouracil
Treg	Regulatory T cells
TAM	tumor-associated macrophages
CCL2	C-C motif chemokine ligand 2
CCL5	CC chemokine ligand 5
CCR2	C-C chemokine receptor type 2
PPARa	Peroxisome proliferator-activated receptor a
PD1	programmed cell death protein 1
PD-L1	Programmed cell death ligand-1
CTLA-4	Cytotoxic T-lymphocyte-associated protein 4
ICAM-1	Intercellular adhesion molecule 1
HIF-1a	Hypoxia-inducible factor-1a
CAFs	Cancer-associated fibroblasts
ECM	extracellular matrix
CXCL13	chemokine ligand 13
CXCR5	chemokine receptor 5
REDOX	reduction/oxidation
GLUT1	glucose transporter type 1
LDHA	lactate dehydrogenase A
PKM2	M2 isoform of pyruvate kinase
TCA cycle	the tricarboxylic acid cycle
a-KG	a-ketoglutarate
PHGDH	phosphoglycerate dehydrogenase
GSH	Glutathione
VEGFA	vascular endothelial growth factor A
HUVEC	human umbilical vein endothelial cells
VEGFR	vascular endothelial growth factor receptor
miRSC	miRNA induced silencing complex
TGF-b	transforming growth factor-b
4EBP-1	eukaryotic translation initiation factor 4E binding protein

## References

[1] Zhou DD Liu XF Lu CW Pant OP Liu XD . Long non-coding RNA PVT1: Emerging biomarker in digestive system cancer. Cell Prolif (2017) 50(6):e12398. doi: 10.1111/cpr.12398 PMC6529066 29027279

[2] Yang S Liu T Cheng Y Bai Y Liang G . Immune cell infiltration as a biomarker for the diagnosis and prognosis of digestive system cancer. Cancer Sci (2019) 110:3639–49. doi: 10.1111/cas.14216 PMC6890448 31605436

[3] Feng W Su Z Yin Q Zong W Shen X Ju S . ncRNAs associated with drug resistance and the therapy of digestive system neoplasms. J Cell Physiol (2019) 234:19143–57. doi: 10.1002/jcp.28551 30941775

[4] Wang J Ma X Si H Ma Z Ma Y Wang J . Role of long non-coding RNA H19 in therapy resistance of digestive system cancers. Mol Med (2021) 27:1. doi: 10.1186/s10020-020-00255-2 33402118 PMC7786989

[5] Sun Z Chen C Su Y Wang W Yang S Zhou Q . Regulatory mechanisms and clinical perspectives of circRNA in digestive system neoplasms. J Cancer (2019) 10:2885–91. doi: 10.7150/jca.31167 PMC6590048 31281465

[6] Zeng D Li M Zhou R Zhang J Sun H Shi M . Tumor microenvironment characterization in gastric cancer identifies prognostic and immunotherapeutically relevant gene signatures. Cancer Immunol Res (2019) 7:737–50. doi: 10.1158/2326-6066.CIR-18-0436 30842092

[7] Zeng D Wu J Luo H Li Y Xiao J Peng J . Tumor microenvironment evaluation promotes precise checkpoint immunotherapy of advanced gastric cancer. J Immunother Cancer (2021) 9(8):e002467. doi: 10.1136/jitc-2021-002467 34376552 PMC8356190

[8] Tang XH Guo T Gao XY Wu XL Xing XF Ji JF . Exosome-derived noncoding RNAs in gastric cancer: Functions and clinical applications. Mol Cancer (2021) 20:99. doi: 10.1186/s12943-021-01396-6 34330299 PMC8323226

[9] Xue C Gu X Bao Z Su Y Lu J Li L . The mechanism underlying the ncRNA dysregulation pattern in hepatocellular carcinoma and its tumor microenvironment. Front Immunol (2022) 13:847728. doi: 10.3389/fimmu.2022.847728 35281015 PMC8904560

[10] Ho WJ Jaffee EM Zheng L . The tumour microenvironment in pancreatic cancer - clinical challenges and opportunities. Nat Rev Clin Oncol (2020) 17:527–40. doi: 10.1038/s41571-020-0363-5 PMC7442729 32398706

[11] Laplane L Duluc D Bikfalvi A Larmonier N Pradeu T . Beyond the tumour microenvironment. Int J Cancer (2019) 145:2611–8. doi: 10.1002/ijc.32343 PMC6766895 30989643

[12] Hussen BM Honarmand Tamizkar K Hidayat HJ Taheri M Ghafouri-Fard S . The role of circular RNAs in the development of hepatocellular carcinoma. Pathol Res Pract (2021) 223:153495. doi: 10.1016/j.prp.2021.153495 34051512

[13] Ghafouri-Fard S Hussen BM Taheri M Ayatollahi SA . Emerging role of circular RNAs in breast cancer. Pathol Res Pract (2021) 223:153496. doi: 10.1016/j.prp.2021.153496 34052769

[14] Ghafouri-Fard S Najafi S Hussen BM Basiri A Hidayat HJ Taheri M . The role of circular RNAs in the carcinogenesis of bladder cancer. Front Oncol (2022) 12:801842. doi: 10.3389/fonc.2022.801842 35296022 PMC8918517

[15] Ghafouri-Fard S Taheri M Hussen BM Vafaeimanesh J Abak A Vafaee R . Function of circular RNAs in the pathogenesis of colorectal cancer. BioMed Pharmacother (2021) 140:111721. doi: 10.1016/j.biopha.2021.111721 34015582

[16] Wu P Mo Y Peng M Tang T Zhong Y Deng X . Emerging role of tumor-related functional peptides encoded by lncRNA and circRNA. Mol Cancer (2020) 19:22. doi: 10.1186/s12943-020-1147-3 32019587 PMC6998289

[17] Tang L Li P Jang M Zhu W . Circular RNAs and cardiovascular regeneration. Front Cardiovasc Med (2021) 8:672600. doi: 10.3389/fcvm.2021.672600 33928139 PMC8076501

[18] Yu T Wang Y Fan Y Fang N Wang T Xu T . CircRNAs in cancer metabolism: a review. J Hematol Oncol (2019) 12:90. doi: 10.1186/s13045-019-0776-8 31484561 PMC6727394

[19] Wang CC Han CD Zhao Q Chen X . Circular RNAs and complex diseases: from experimental results to computational models. Brief Bioinform (2021) 22(6): bbab286. doi: 10.1093/bib/bbab286 34329377 PMC8575014

[20] Patop IL Kadener S . circRNAs in cancer. Curr Opin Genet Dev (2018) 48:121–7. doi: 10.1016/j.gde.2017.11.007 PMC5877416 29245064

[21] Qi L Wang W Zhao G Jiang H Zhang Y Zhao D . Circular RNA circitga7 accelerates glioma progression *via* miR-34a-5p/VEGFA axis. Aging (Albany NY) (2021) 13:13138–52. doi: 10.18632/aging.202996 PMC8148479 33962397

[22] Zhu J Luo Y Zhao Y Kong Y Zheng H Li Y . circEHBP1 promotes lymphangiogenesis and lymphatic metastasis of bladder cancer *via* miR-130a-3p/TGFβR1/VEGF-D signaling. Mol Ther (2021) 29:1838–52. doi: 10.1016/j.ymthe.2021.01.031 PMC8116613 33545359

[23] Ma Y Li Z Ma D Guo J Sun W . Hsa_circ_0003195 as a biomarker for diagnosis and prognosis of gastric cancer. Int J Clin Oncol (2022) 27:354–61. doi: 10.1007/s10147-021-02073-w 34773528

[24] Zhang W Zheng M Kong S Li X Meng S Wang X . Circular RNA hsa_circ_0007507 may serve as a biomarker for the diagnosis and prognosis of gastric cancer. Front Oncol (2021) 11:699625. doi: 10.3389/fonc.2021.699625 34595108 PMC8477006

[25] Yan J Shao Y Lu H Ye Q Ye G Guo J . Hsa_circ_0001020 serves as a potential biomarker for gastric cancer screening and prognosis. Dig Dis Sci (2021) 67(8):3753–62. doi: 10.1007/s10620-021-07211-y 34424459

[26] Xu Y Kong S Qin X Ju S . Comprehensive assessment of plasma Circ_0004771 as a novel diagnostic and dynamic monitoring biomarker in gastric cancer. Onco Targets Ther (2020) 13:10063–74. doi: 10.2147/OTT.S263536 PMC7549879 33116589

[27] Chen LL Yang L . Regulation of circRNA biogenesis. RNA Biol (2015) 12:381–8. doi: 10.1080/15476286.2015.1020271 PMC4615371 25746834

[28] Prats AC David F Diallo LH Roussel E Tatin F Garmy-Susini B . Circular RNA, the key for translation. Int J Mol Sci (2020) 21(22):8591. doi: 10.3390/ijms21228591 PMC7697609 33202605

[29] Lee KM Chen CJ Shih SR . Regulation mechanisms of viral IRES-driven translation. Trends Microbiol (2017) 25:546–61. doi: 10.1016/j.tim.2017.01.010 28242053

[30] Johnson AG Grosely R Petrov AN Puglisi JD . Dynamics of IRES-mediated translation. Philos Trans R Soc Lond B Biol Sci (2017) 372(1716):20160177. doi: 10.1098/rstb.2016.0177 28138065 PMC5311923

[31] Martinez-Salas E Francisco-Velilla R Fernandez-Chamorro J Embarek AM . Insights into structural and mechanistic features of viral IRES elements. Front Microbiol (2017) 8:2629. doi: 10.3389/fmicb.2017.02629 29354113 PMC5759354

[32] Yang Y Wang Z . IRES-mediated cap-independent translation, a path leading to hidden proteome. J Mol Cell Biol (2019) 11:911–9. doi: 10.1093/jmcb/mjz091 PMC6884710 31504667

[33] Huang A Zheng H Wu Z Chen M Huang Y . Circular RNA-protein interactions: functions, mechanisms, and identification. Theranostics (2020) 10:3503–17. doi: 10.7150/thno.42174 PMC7069073 32206104

[34] Liu ZX Li LM Sun HL Liu SM . Link between m6A modification and cancers. Front Bioeng Biotechnol (2018) 6:89. doi: 10.3389/fbioe.2018.00089 30062093 PMC6055048

[35] Jiang X Liu B Nie Z Duan L Xiong Q Jin Z . The role of m6A modification in the biological functions and diseases. Signal Transduct Target Ther (2021) 6:74. doi: 10.1038/s41392-020-00450-x 33611339 PMC7897327

[36] Ma Z Ji J . N6-methyladenosine (m6A) RNA modification in cancer stem cells. Stem Cells (2020) 38(12):1511–9. doi: 10.1002/stem.3279 32985068

[37] Kristensen LS Andersen MS Stagsted LVW Ebbesen KK Hansen TB Kjems J . The biogenesis, biology and characterization of circular RNAs. Nat Rev Genet (2019) 20:675–91. doi: 10.1038/s41576-019-0158-7 31395983

[38] Sinha T Panigrahi C Das D Chandra Panda A . Circular RNA translation, a path to hidden proteome. Wiley Interdiscip Rev RNA (2022) 13:e1685. doi: 10.1002/wrna.1685 34342387 PMC7613019

[39] Tran AM Chalbatani GM Berland L Cruz De Los Santos M Raj P Jalali SA . A new world of biomarkers and therapeutics for female reproductive system and breast cancers: Circular RNAs. Front Cell Dev Biol (2020) 8:50. doi: 10.3389/fcell.2020.00050 32211400 PMC7075436

[40] Hsiao KY Sun HS Tsai SJ . Circular RNA - new member of noncoding RNA with novel functions. Exp Biol Med (Maywood) (2017) 242:1136–41. doi: 10.1177/1535370217708978 PMC5478007 28485684

[41] Salzman J . Circular RNA expression: Its potential regulation and function. Trends Genet (2016) 32:309–16. doi: 10.1016/j.tig.2016.03.002 PMC4948998 27050930

[42] Patop IL Wüst S Kadener S . Past, present, and future of circRNAs. EMBO J (2019) 38:e100836. doi: 10.15252/embj.2018100836 31343080 PMC6694216

[43] Barrett SP Salzman J . Circular RNAs: Analysis, expression and potential functions. Development (2016) 143:1838–47. doi: 10.1242/dev.128074 PMC4920157 27246710

[44] Panda AC Grammatikakis I Munk R Gorospe M Abdelmohsen K . Emerging roles and context of circular RNAs. Wiley Interdiscip Rev RNA (2017) 8(2):10.1002. doi: 10.1002/wrna.1386 PMC5315638 27612318

[45] Meng S Zhou H Feng Z Xu Z Tang Y Li P . CircRNA: functions and properties of a novel potential biomarker for cancer. Mol Cancer (2017) 16:94. doi: 10.1186/s12943-017-0663-2 28535767 PMC5440908

[46] Liu CX Li X Nan F Jiang S Gao X Guo SK . Structure and degradation of circular RNAs regulate PKR activation in innate immunity. Cell (2019) 177:865–880.e821. doi: 10.1016/j.cell.2019.03.046 31031002

[47] Guria A Sharma P Natesan S Pandi G . Circular RNAs-the road less traveled. Front Mol Biosci (2019) 6:146. doi: 10.3389/fmolb.2019.00146 31998746 PMC6965350

[48] Dori M Bicciato S . Integration of bioinformatic predictions and experimental data to identify circRNA-miRNA associations. Genes (Basel) (2019) 10(9):642. doi: 10.3390/genes10090642 PMC6769881 31450634

[49] Chen Y Yang F Fang E Xiao W Mei H Li H . Circular RNA circAGO2 drives cancer progression through facilitating HuR-repressed functions of AGO2-miRNA complexes. Cell Death Differ (2019) 26:1346–64. doi: 10.1038/s41418-018-0220-6 PMC6748083 30341421

[50] Pantazopoulou VI Delis AD Georgiou S Pagakis SN Filippa V Dragona E . AGO2 localizes to cytokinetic protrusions in a p38-dependent manner and is needed for accurate cell division. Commun Biol (2021) 4:726. doi: 10.1038/s42003-021-02130-0 34117353 PMC8196063

[51] Sheu-Gruttadauria J MacRae IJ . Phase transitions in the assembly and function of human miRISC. Cell (2018) 173:946–957.e916. doi: 10.1016/j.cell.2018.02.051 29576456 PMC5935535

[52] Pan Z Li GF Sun ML Xie L Liu D Zhang Q . MicroRNA-1224 splicing CircularRNA-Filip1l in an Ago2-dependent manner regulates chronic inflammatory pain *via* targeting Ubr5. J Neurosci (2019) 39:2125–43. doi: 10.1523/JNEUROSCI.1631-18.2018 PMC6507086 30651325

[53] Marzec M . New insights into the function of mammalian Argonaute2. PloS Genet (2020) 16:e1009058. doi: 10.1371/journal.pgen.1009058 33180792 PMC7660459

[54] Zhang L Hou C Chen C Guo Y Yuan W Yin D . The role of N(6)-methyladenosine (m(6)A) modification in the regulation of circRNAs. Mol Cancer (2020) 19:105. doi: 10.1186/s12943-020-01224-3 32522202 PMC7285594

[55] Wang X Ma R Zhang X Cui L Ding Y Shi W . Crosstalk between N6-methyladenosine modification and circular RNAs: Current understanding and future directions. Mol Cancer (2021) 20:121. doi: 10.1186/s12943-021-01415-6 34560891 PMC8461955

[56] An Y Duan H . The role of m6A RNA methylation in cancer metabolism. Mol Cancer (2022) 21:14. doi: 10.1186/s12943-022-01500-4 35022030 PMC8753874

[57] Park OH Ha H Lee Y Boo SH Kwon DH Song HK . Endoribonucleolytic cleavage of m(6)A-containing RNAs by RNase P/MRP complex. Mol Cell (2019) 74:494–507.e498. doi: 10.1016/j.molcel.2019.02.034 30930054

[58] Liu CX Chen LL . Circular RNAs: Characterization, cellular roles, and applications. Cell (2022) 185:2016–34. doi: 10.1016/j.cell.2022.04.021 35584701

[59] Wang C Tan S Li J Liu WR Peng Y Li W . CircRNAs in lung cancer - biogenesis, function and clinical implication. Cancer Lett (2020) 492:106–15. doi: 10.1016/j.canlet.2020.08.013 32860847

[60] Misir S Wu N Yang BB . Specific expression and functions of circular RNAs. Cell Death Differ (2022) 29:481–91. doi: 10.1038/s41418-022-00948-7 PMC8901656 35169296

[61] Rossi F Beltran M Damizia M Grelloni C Colantoni A Setti A . Circular RNA ZNF609/CKAP5 mRNA interaction regulates microtubule dynamics and tumorigenicity. Mol Cell (2022) 82:75–89.e79. doi: 10.1016/j.molcel.2021.11.032 34942120 PMC8751636

[62] Guo L Jia L Luo L Xu X Xiang Y Ren Y . Critical roles of circular RNA in tumor metastasis *via* acting as a sponge of miRNA/isomiR. Int J Mol Sci (2022) 23(13):7024. doi: 10.3390/ijms23137024 35806027 PMC9267010

[63] Qiu S Li B Xia Y Xuan Z Li Z Xie L . CircTHBS1 drives gastric cancer progression by increasing INHBA mRNA expression and stability in a ceRNA- and RBP-dependent manner. Cell Death Dis (2022) 13:266. doi: 10.1038/s41419-022-04720-0 35338119 PMC8949653

[64] Chen J Gu J Tang M Liao Z Tang R Zhou L . Regulation of cancer progression by circRNA and functional proteins. J Cell Physiol (2022) 237:373–88. doi: 10.1002/jcp.30608 34676546

[65] Chen J Yang J Fei X Wang X Wang K . CircRNA ciRS-7: a novel oncogene in multiple cancers. Int J Biol Sci (2021) 17:379–89. doi: 10.7150/ijbs.54292 PMC7757028 33390857

[66] Hansen TB Kjems J Damgaard CK . Circular RNA and miR-7 in cancer. Cancer Res (2013) 73:5609–12. doi: 10.1158/0008-5472.CAN-13-1568 24014594

[67] Li RC Ke S Meng FK Lu J Zou XJ He ZG . CiRS-7 promotes growth and metastasis of esophageal squamous cell carcinoma *via* regulation of miR-7/HOXB13. Cell Death Dis (2018) 9:838. doi: 10.1038/s41419-018-0852-y 30082829 PMC6079012

[68] Pan H Li T Jiang Y Pan C Ding Y Huang Z . Overexpression of circular RNA ciRS-7 abrogates the tumor suppressive effect of miR-7 on gastric cancer *via* PTEN/PI3K/AKT signaling pathway. J Cell Biochem (2018) 119:440–6. doi: 10.1002/jcb.26201 28608528

[69] Chen X Lu Y . Circular RNA: Biosynthesis. Vitro Front Bioeng Biotechnol (2021) 9:787881. doi: 10.3389/fbioe.2021.787881 34917603 PMC8670002

[70] Bose R Ain R . Regulation of transcription by circular RNAs. Adv Exp Med Biol (2018) 1087:81–94. doi: 10.1007/978-981-13-1426-1_7 30259359

[71] Pamudurti NR Patop IL Krishnamoorthy A Bartok O Maya R Lerner N . circMbl functions in cis and in trans to regulate gene expression and physiology in a tissue-specific fashion. Cell Rep (2022) 39:110740. doi: 10.1016/j.celrep.2022.110740 35476987 PMC9352392

[72] Ashwal-Fluss R Meyer M Pamudurti NR Ivanov A Bartok O Hanan M . circRNA biogenesis competes with pre-mRNA splicing. Mol Cell (2014) 56:55–66. doi: 10.1016/j.molcel.2014.08.019 25242144

[73] Du WW Yang W Liu E Yang Z Dhaliwal P Yang BB . Foxo3 circular RNA retards cell cycle progression *via* forming ternary complexes with p21 and CDK2. Nucleic Acids Res (2016) 44:2846–58. doi: 10.1093/nar/gkw027 PMC4824104 26861625

[74] Shi Y Jia X Xu J . The new function of circRNA: translation. Clin Transl Oncol (2020) 22:2162–9. doi: 10.1007/s12094-020-02371-1 32449127

[75] Kong S Tao M Shen X Ju S . Translatable circRNAs and lncRNAs: Driving mechanisms and functions of their translation products. Cancer Lett (2020) 483:59–65. doi: 10.1016/j.canlet.2020.04.006 32360179

[76] Qin S Mao Y Chen X Xiao J Qin Y Zhao L . The functional roles, cross-talk and clinical implications of m6A modification and circRNA in hepatocellular carcinoma. Int J Biol Sci (2021) 17:3059–79. doi: 10.7150/ijbs.62767 PMC8375232 34421350

[77] Chen LL . The expanding regulatory mechanisms and cellular functions of circular RNAs. Nat Rev Mol Cell Biol (2020) 21:475–90. doi: 10.1038/s41580-020-0243-y 32366901

[78] Xu X Zhang J Tian Y Gao Y Dong X Chen W . CircRNA inhibits DNA damage repair by interacting with host gene. Mol Cancer (2020) 19:128. doi: 10.1186/s12943-020-01246-x 32838810 PMC7446195

[79] Mehta SL Dempsey RJ Vemuganti R . Role of circular RNAs in brain development and CNS diseases. Prog Neurobiol (2020) 186:101746. doi: 10.1016/j.pneurobio.2020.101746 31931031 PMC7024016

[80] Cao P Ma B Sun D Zhang W Qiu J Qin L . hsa_circ_0003410 promotes hepatocellular carcinoma progression by increasing the ratio of M2/M1 macrophages through the miR-139-3p/CCL5 axis. Cancer Sci (2022) 113:634–47. doi: 10.1111/cas.15238 PMC8819332 34890089

[81] Liu B Tian Y Chen M Shen H Xia J Nan J . CircUBAP2 promotes MMP9-mediated oncogenic effect *via* sponging miR-194-3p in hepatocellular carcinoma. Front Cell Dev Biol (2021) 9:675043. doi: 10.3389/fcell.2021.675043 34239873 PMC8258265

[82] Li J Qin X Wu R Wan L Zhang L Liu R . Circular RNA circFBXO11 modulates hepatocellular carcinoma progress and oxaliplatin resistance through miR-605/FOXO3/ABCB1 axis. J Cell Mol Med (2020) 24:5152–61. doi: 10.1111/jcmm.15162 PMC7205830 32222024

[83] Wang X Sheng W Xu T Xu J Gao R Zhang Z . CircRNA hsa_circ_0110102 inhibited macrophage activation and hepatocellular carcinoma progression *via* miR-580-5p/PPARα/CCL2 pathway. Aging (Albany NY) (2021) 13:11969–87. doi: 10.18632/aging.202900 PMC8109088 33891564

[84] Xu J Wan Z Tang M Lin Z Jiang S Ji L . N(6)-methyladenosine-modified CircRNA-SORE sustains sorafenib resistance in hepatocellular carcinoma by regulating β-catenin signaling. Mol Cancer (2020) 19:163. doi: 10.1186/s12943-020-01281-8 33222692 PMC7681956

[85] Shi M Li ZY Zhang LM Wu XY Xiang SH Wang YG . Hsa_circ_0007456 regulates the natural killer cell-mediated cytotoxicity toward hepatocellular carcinoma *via* the miR-6852-3p/ICAM-1 axis. Cell Death Dis (2021) 12:94. doi: 10.1038/s41419-020-03334-8 33462208 PMC7814008

[86] Liu H Lan T Li H Xu L Chen X Liao H . Circular RNA circDLC1 inhibits MMP1-mediated liver cancer progression *via* interaction with HuR. Theranostics (2021) 11:1396–411. doi: 10.7150/thno.53227 PMC7738888 33391541

[87] Liu G Sun J Yang ZF Zhou C Zhou PY Guan RY . Cancer-associated fibroblast-derived CXCL11 modulates hepatocellular carcinoma cell migration and tumor metastasis through the circUBAP2/miR-4756/IFIT1/3 axis. Cell Death Dis (2021) 12:260. doi: 10.1038/s41419-021-03545-7 33707417 PMC7952559

[88] Xu G Zhang P Liang H Xu Y Shen J Wang W . Circular RNA hsa_circ_0003288 induces EMT and invasion by regulating hsa_circ_0003288/miR-145/PD-L1 axis in hepatocellular carcinoma. Cancer Cell Int (2021) 21:212. doi: 10.1186/s12935-021-01902-2 33858418 PMC8048300

[89] Lu JC Zhang PF Huang XY Guo XJ Gao C Zeng HY . Amplification of spatially isolated adenosine pathway by tumor-macrophage interaction induces anti-PD1 resistance in hepatocellular carcinoma. J Hematol Oncol (2021) 14:200. doi: 10.1186/s13045-021-01207-x 34838121 PMC8627086

[90] Zhang PF Gao C Huang XY Lu JC Guo XJ Shi GM . Cancer cell-derived exosomal circUHRF1 induces natural killer cell exhaustion and may cause resistance to anti-PD1 therapy in hepatocellular carcinoma. Mol Cancer (2020) 19:110. doi: 10.1186/s12943-020-01222-5 32593303 PMC7320583

[91] Wang Y Gao R Li J Tang S Li S Tong Q . Downregulation of hsa_circ_0074854 suppresses the migration and invasion in hepatocellular carcinoma *via* interacting with HuR and *via* suppressing exosomes-mediated macrophage M2 polarization. Int J Nanomedicine (2021) 16:2803–18. doi: 10.2147/IJN.S284560 PMC8052130 33880025

[92] Zhou H Chen Y . CircRNA has_circ_0001806 promotes hepatocellular carcinoma progression *via* the miR-193a-5p/MMP16 pathway. Braz J Med Biol Res (2021) 54:e11459. doi: 10.1590/1414-431x2021e11459 34730679 PMC8555451

[93] Hou JP Men XB Yang LY Han EK Han CQ Liu LB . CircCCT3 acts as a sponge of miR-613 to promote tumor growth of pancreatic cancer through regulating VEGFA/VEGFR2 signaling. Balkan Med J (2021) 38:229–38. doi: 10.5152/balkanmedj.2021.21145 PMC8880967 34274912

[94] Zhang R Zhu W Ma C Ai K . Silencing of circRNA circ_0001666 represses EMT in pancreatic cancer through upregulating miR-1251 and downregulating SOX4. Front Mol Biosci (2021) 8:684866. doi: 10.3389/fmolb.2021.684866 34055896 PMC8155604

[95] Hong C Lishan W Peng X Zhengqing L Yang Y Fangfang H . Hsa_circ_0074298 promotes pancreatic cancer progression and resistance to gemcitabine by sponging miR-519 to target SMOC. J Cancer (2022) 13:34–50. doi: 10.7150/jca.62927 34976169 PMC8692684

[96] Kong Y Li Y Luo Y Zhu J Zheng H Gao B . circNFIB1 inhibits lymphangiogenesis and lymphatic metastasis *via* the miR-486-5p/PIK3R1/VEGF-C axis in pancreatic cancer. Mol Cancer (2020) 19:82. doi: 10.1186/s12943-020-01205-6 32366257 PMC7197141

[97] Yu S Wang M Zhang H Guo X Qin R . Circ_0092367 inhibits EMT and gemcitabine resistance in pancreatic cancer *via* regulating the miR-1206/ESRP1 axis. Genes (Basel) (2021) 12(11):1701. doi: 10.3390/genes12111701 34828307 PMC8622583

[98] Zeng Z Zhao Y Chen Q Zhu S Niu Y Ye Z . Hypoxic exosomal HIF-1α-stabilizing circZNF91 promotes chemoresistance of normoxic pancreatic cancer cells *via* enhancing glycolysis. Oncogene (2021) 40:5505–17. doi: 10.1038/s41388-021-01960-w 34294845

[99] Xie M Yu T Jing X Ma L Fan Y Yang F . Exosomal circSHKBP1 promotes gastric cancer progression *via* regulating the miR-582-3p/HUR/VEGF axis and suppressing HSP90 degradation. Mol Cancer (2020) 19:112. doi: 10.1186/s12943-020-01208-3 32600329 PMC7322843

[100] Lu J Wang YH Yoon C Huang XY Xu Y Xie JW . Circular RNA circ-RanGAP1 regulates VEGFA expression by targeting miR-877-3p to facilitate gastric cancer invasion and metastasis. Cancer Lett (2020) 471:38–48. doi: 10.1016/j.canlet.2019.11.038 31811909

[101] Peng L Sang H Wei S Li Y Jin D Zhu X . circCUL2 regulates gastric cancer malignant transformation and cisplatin resistance by modulating autophagy activation *via* miR-142-3p/ROCK2. Mol Cancer (2020) 19:156. doi: 10.1186/s12943-020-01270-x 33153478 PMC7643398

[102] Ren J Pan G Yang J Xu N Zhang Q Li W . Circ_0000620 acts as an oncogenic factor in gastric cancer through regulating MMP2 expression *via* sponging miR-671-5p. J Biol Res (Thessalon) (2021) 28:23. doi: 10.1186/s40709-021-00154-5 34972532 PMC8720221

[103] Yao W Guo P Mu Q Wang Y . Exosome-derived circ-PVT1 contributes to cisplatin resistance by regulating autophagy, invasion, and apoptosis *Via* miR-30a-5p/YAP1 axis in gastric cancer cells. Cancer Biother Radiopharm (2021) 36:347–59. doi: 10.1089/cbr.2020.3578 32799541

[104] Zhang R Zhao H Yuan H Wu J Liu H Sun S . CircARVCF contributes to cisplatin resistance in gastric cancer by altering miR-1205 and FGFR1. Front Genet (2021) 12:767590. doi: 10.3389/fgene.2021.767590 34899853 PMC8656457

[105] Liu S Wu M Peng M . Circ_0000260 regulates the development and deterioration of gastric adenocarcinoma with cisplatin resistance by upregulating MMP11 *via* targeting MiR-129-5p. Cancer Manag Res (2020) 12:10505–19. doi: 10.2147/CMAR.S272324 PMC7591103 33122949

[106] Sun G Li Z He Z Wang W Wang S Zhang X . Circular RNA MCTP2 inhibits cisplatin resistance in gastric cancer by miR-99a-5p-mediated induction of MTMR3 expression. J Exp Clin Cancer Res (2020) 39:246. doi: 10.1186/s13046-020-01758-w 33198772 PMC7670601

[107] Du Y Zhang JY Gong LP Feng ZY Wang D Pan YH . Hypoxia-induced ebv-circLMP2A promotes angiogenesis in EBV-associated gastric carcinoma through the KHSRP/VHL/HIF1α/VEGFA pathway. Cancer Lett (2022) 526:259–72. doi: 10.1016/j.canlet.2021.11.031 34863886

[108] Liu J Dai X Guo X Cheng A Mac SM Wang Z . Circ-OXCT1 suppresses gastric cancer EMT and metastasis by attenuating TGF-β pathway through the circ-OXCT1/miR-136/SMAD4 axis. Onco Targets Ther (2020) 13:3987–98. doi: 10.2147/OTT.S239789 PMC7236241 32523351

[109] Shen D Zhao H Zeng P Song J Yang Y Gu X . Circular RNA hsa_circ_0005556 accelerates gastric cancer progression by sponging miR-4270 to increase MMP19 expression. J Gastric Cancer (2020) 20:300–12. doi: 10.5230/jgc.2020.20.e28 PMC7521983 33024586

[110] Chen W Ji Y . CircC6orf132 facilitates proliferation, migration, invasion, and glycolysis of gastric cancer cells under hypoxia by acting on the miR-873-5p/PRKAA1 axis. Front Genet (2021) 12:636392. doi: 10.3389/fgene.2021.636392 34659329 PMC8514671

[111] Tang J Zhu H Lin J Wang H . Knockdown of Circ_0081143 mitigates hypoxia-induced migration, invasion, and EMT in gastric cancer cells through the miR-497-5p/EGFR axis. Cancer Biother Radiopharm (2021) 36:333–46. doi: 10.1089/cbr.2019.3512 32678674

[112] Yang H Zhang H Yang Y Wang X Deng T Liu R . Hypoxia induced exosomal circRNA promotes metastasis of colorectal cancer *via* targeting GEF-H1/RhoA axis. Theranostics (2020) 10:8211–26. doi: 10.7150/thno.44419 PMC7381736 32724467

[113] Wang Q Shi L Shi K Yuan B Cao G Kong C . CircCSPP1 functions as a ceRNA to promote colorectal carcinoma cell EMT and liver metastasis by upregulating COL1A1. Front Oncol (2020) 10:850. doi: 10.3389/fonc.2020.00850 32612946 PMC7308451

[114] Liu X Liu Y Liu Z Lin C Meng F Xu L . CircMYH9 drives colorectal cancer growth by regulating serine metabolism and redox homeostasis in a p53-dependent manner. Mol Cancer (2021) 20:114. doi: 10.1186/s12943-021-01412-9 34496888 PMC8424912

[115] Jin L Han C Zhai T Zhang X Chen C Lian L . Circ_0030998 promotes tumor proliferation and angiogenesis by sponging miR-567 to regulate VEGFA in colorectal cancer. Cell Death Discovery (2021) 7:160. doi: 10.1038/s41420-021-00544-7 34226531 PMC8257860

[116] Yang K Zhang J Bao C . Exosomal circEIF3K from cancer-associated fibroblast promotes colorectal cancer (CRC) progression *via* miR-214/PD-L1 axis. BMC Cancer (2021) 21:933. doi: 10.1186/s12885-021-08669-9 34412616 PMC8375187

[B117] Gu C Lu H Qian Z . Matrine reduces the secretion of exosomal circSLC7A6 from cancer-associated fibroblast to inhibit tumorigenesis of colorectal cancer by regulating CXCR5. Biochem Biophys Res Commun (2020) 527:638–45. doi: 10.1016/j.bbrc.2020.04.142 32423804

[118] He X Ma J Zhang M Cui J Yang H . Circ_0007031 enhances tumor progression and promotes 5-fluorouracil resistance in colorectal cancer through regulating miR-133b/ABCC5 axis. Cancer biomark (2020) 29:531–42. doi: 10.3233/CBM-200023 PMC12662547 32865180

[119] Wang X Zhang H Yang H Bai M Ning T Deng T . Exosome-delivered circRNA promotes glycolysis to induce chemoresistance through the miR-122-PKM2 axis in colorectal cancer. Mol Oncol (2020) 14:539–55. doi: 10.1002/1878-0261.12629 PMC7053238 31901148

[120] Sun S Li C Cui K Liu B Zhou M Cao Y . Hsa_circ_0062682 promotes serine metabolism and tumor growth in colorectal cancer by regulating the miR-940/PHGDH axis. Front Cell Dev Biol (2021) 9:770006. doi: 10.3389/fcell.2021.770006 34957102 PMC8692793

[121] Dai J Zhuang Y Tang M Qian Q Chen JP . CircRNA UBAP2 facilitates the progression of colorectal cancer by regulating miR-199a/VEGFA pathway. Eur Rev Med Pharmacol Sci (2020) 24:7963–71. doi: 10.26355/eurrev_202008_22479 32767322

[122] Chen LY Wang L Ren YX Pang Z Liu Y Sun XD . The circular RNA circ-ERBIN promotes growth and metastasis of colorectal cancer by miR-125a-5p and miR-138-5p/4EBP-1 mediated cap-independent HIF-1α translation. Mol Cancer (2020) 19:164. doi: 10.1186/s12943-020-01272-9 33225938 PMC7682012

[123] Dai W Zhai X Chen Y Bai Y Deng H Zhu R . CircMMP1 promotes colorectal cancer growth and metastasis by sponging miR-1238 and upregulating MMP family expression. Ann Transl Med (2021) 9:1341. doi: 10.21037/atm-21-3930 34532478 PMC8422139

[124] Zheng X Ma YF Zhang XR Li Y Zhao HH Han SG . Circ_0056618 promoted cell proliferation, migration and angiogenesis through sponging with miR-206 and upregulating CXCR4 and VEGF-a in colorectal cancer. Eur Rev Med Pharmacol Sci (2020) 24:4190–202. doi: 10.26355/eurrev_202004_20999 32373955

[125] Feng J Li Z Li L Xie H Lu Q He X . Hypoxia−induced circCCDC66 promotes the tumorigenesis of colorectal cancer *via* the miR−3140/autophagy pathway. Int J Mol Med (2020) 46:1973–82. doi: 10.3892/ijmm.2020.4747 PMC7595663 33125087

[126] Xu YJ Zhao JM Gao C Ni XF Wang W Hu WW . Hsa_circ_0136666 activates treg-mediated immune escape of colorectal cancer *via* miR-497/PD-L1 pathway. Cell Signal (2021) 86:110095. doi: 10.1016/j.cellsig.2021.110095 34320370

[127] Liang Z Zhao B Hou J Zheng J Xin G . CircRNA circ-OGDH (hsa_circ_0003340) acts as a ceRNA to regulate glutamine metabolism and esophageal squamous cell carcinoma progression by the miR-615-5p/PDX1 axis. Cancer Manag Res (2021) 13:3041–53. doi: 10.2147/CMAR.S290088 PMC8039021 33854374

[128] Guo J Su Y Zhang M . Circ_0000140 restrains the proliferation, metastasis and glycolysis metabolism of oral squamous cell carcinoma through upregulating CDC73 *via* sponging miR-182-5p. Cancer Cell Int (2020) 20:407. doi: 10.1186/s12935-020-01501-7 32863766 PMC7448321

[129] Qian C Chen S Li S Wang Y Yao J . Circ_0000003 regulates glutamine metabolism and tumor progression of tongue squamous cell carcinoma *via* the miR−330−3p/GLS axis. Oncol Rep (2021) 45(4):45. doi: 10.3892/or.2021.7996 33649795 PMC7934215

[130] Jia Z Jia J Yao L Li Z . Crosstalk of exosomal non-coding RNAs in the tumor microenvironment: Novel frontiers. Front Immunol (2022) 13:900155. doi: 10.3389/fimmu.2022.900155 35663957 PMC9162146

[131] Natua S Dhamdhere SG Mutnuru SA Shukla S . Interplay within tumor microenvironment orchestrates neoplastic RNA metabolism and transcriptome diversity. Wiley Interdiscip Rev RNA (2022) 13:e1676. doi: 10.1002/wrna.1676 34109748

[132] Khan SU Khan MU Khan MI Abraham FA Khan A Gao S . Role of circular RNAs in disease progression and diagnosis of cancers: An overview of recent advanced insights. Int J Biol Macromol (2022) 220:973–84. doi: 10.1016/j.ijbiomac.2022.08.085 35977596

[133] Salmaninejad A Valilou SF Soltani A Ahmadi S Abarghan YJ Rosengren RJ . Tumor-associated macrophages: role in cancer development and therapeutic implications. Cell Oncol (Dordr) (2019) 42:591–608. doi: 10.1007/s13402-019-00453-z 31144271 PMC12994359

[134] DeNardo DG Ruffell B . Macrophages as regulators of tumour immunity and immunotherapy. Nat Rev Immunol (2019) 19:369–82. doi: 10.1038/s41577-019-0127-6 PMC7339861 30718830

[135] Mehla K Singh PK . Metabolic regulation of macrophage polarization in cancer. Trends Cancer (2019) 5:822–34. doi: 10.1016/j.trecan.2019.10.007 PMC7187927 31813459

[136] Myers KV Pienta KJ Amend SR . Cancer cells and M2 macrophages: Cooperative invasive ecosystem engineers. Cancer Control (2020) 27:1073274820911058. doi: 10.1177/1073274820911058 32129079 PMC7066590

[137] Zhao S Mi Y Guan B Zheng B Wei P Gu Y . Tumor-derived exosomal miR-934 induces macrophage M2 polarization to promote liver metastasis of colorectal cancer. J Hematol Oncol (2020) 13:156. doi: 10.1186/s13045-020-00991-2 33213490 PMC7678301

[138] Liu Q Yang C Wang S Shi D Wei C Song J . Wnt5a-induced M2 polarization of tumor-associated macrophages *via* IL-10 promotes colorectal cancer progression. Cell Commun Signal (2020) 18:51. doi: 10.1186/s12964-020-00557-2 32228612 PMC7106599

[139] Wang D Wang X Si M Yang J Sun S Wu H . Exosome-encapsulated miRNAs contribute to CXCL12/CXCR4-induced liver metastasis of colorectal cancer by enhancing M2 polarization of macrophages. Cancer Lett (2020) 474:36–52. doi: 10.1016/j.canlet.2020.01.005 31931030

[140] Wang P Wang H Huang Q Peng C Yao L Chen H . Exosomes from M1-polarized macrophages enhance paclitaxel antitumor activity by activating macrophages-mediated inflammation. Theranostics (2019) 9:1714–27. doi: 10.7150/thno.30716 PMC6485189 31037133

[141] Vitale I Manic G Coussens LM Kroemer G Galluzzi L . Macrophages and metabolism in the tumor microenvironment. Cell Metab (2019) 30:36–50. doi: 10.1016/j.cmet.2019.06.001 31269428

[142] Zhou Z Jiang R Yang X Guo H Fang S Zhang Y . circRNA mediates silica-induced macrophage activation *Via* HECTD1/ZC3H12A-dependent ubiquitination. Theranostics (2018) 8:575–92. doi: 10.7150/thno.21648 PMC5743568 29290828

[143] Aldinucci D Borghese C Casagrande N . The CCL5/CCR5 axis in cancer progression. Cancers (Basel) (2020) 12(7):1765. doi: 10.3390/cancers12071765 PMC7407580 32630699

[144] Korbecki J Grochans S Gutowska I Barczak K Baranowska-Bosiacka I . CC chemokines in a tumor: A review of pro-cancer and anti-cancer properties of receptors CCR5, CCR6, CCR7, CCR8, CCR9, and CCR10 ligands. Int J Mol Sci (2020) 21(20):7619. doi: 10.3390/ijms21207619 PMC7590012 33076281

[145] Hao Q Vadgama JV Wang P . CCL2/CCR2 signaling in cancer pathogenesis. Cell Commun Signal (2020) 18:82. doi: 10.1186/s12964-020-00589-8 32471499 PMC7257158

[146] Xu M Wang Y Xia R Wei Y Wei X . Role of the CCL2-CCR2 signalling axis in cancer: Mechanisms and therapeutic targeting. Cell Prolif (2021) 54:e13115. doi: 10.1111/cpr.13115 34464477 PMC8488570

[147] Li X Yao W Yuan Y Chen P Li B Li J . Targeting of tumour-infiltrating macrophages *via* CCL2/CCR2 signalling as a therapeutic strategy against hepatocellular carcinoma. Gut (2017) 66:157–67. doi: 10.1136/gutjnl-2015-310514 26452628

[148] Pan Z Zhao R Li B Qi Y Qiu W Guo Q . EWSR1-induced circNEIL3 promotes glioma progression and exosome-mediated macrophage immunosuppressive polarization *via* stabilizing IGF2BP3. Mol Cancer (2022) 21:16. doi: 10.1186/s12943-021-01485-6 35031058 PMC8759291

[149] Zhao S Liu Y He L Li Y Lin K Kang Q . Gallbladder cancer cell-derived exosome-mediated transfer of leptin promotes cell invasion and migration by modulating STAT3-mediated M2 macrophage polarization. Anal Cell Pathol (Amst) (2022) 2022:9994906. doi: 10.1155/2022/9994906 35111566 PMC8803447

[150] Han C Zhang C Wang H Zhao L . Exosome-mediated communication between tumor cells and tumor-associated macrophages: Implications for tumor microenvironment. Oncoimmunology (2021) 10:1887552. doi: 10.1080/2162402X.2021.1887552 33680573 PMC7901554

[151] Wei QT Liu BY Ji HY Lan YF Tang WH Zhou J . Exosome-mediated transfer of MIF confers temozolomide resistance by regulating TIMP3/PI3K/AKT axis in gliomas. Mol Ther Oncolytics (2021) 22:114–28. doi: 10.1016/j.omto.2021.08.004 PMC8413833 34514093

[152] Lei Q Wang D Sun K Wang L Zhang Y . Resistance mechanisms of anti-PD1/PDL1 therapy in solid tumors. Front Cell Dev Biol (2020) 8:672. doi: 10.3389/fcell.2020.00672 32793604 PMC7385189

[153] Zhang Q Tang L Zhou Y He W Li W . Immune checkpoint inhibitor-associated pneumonitis in non-small cell lung cancer: Current understanding in characteristics, diagnosis, and management. Front Immunol (2021) 12:663986. doi: 10.3389/fimmu.2021.663986 34122422 PMC8195248

[154] Perdigoto AL Kluger H Herold KC . Adverse events induced by immune checkpoint inhibitors. Curr Opin Immunol (2021) 69:29–38. doi: 10.1016/j.coi.2021.02.002 33640598 PMC8122053

[155] Li Z Li Y Gao J Fu Y Hua P Jing Y . The role of CD47-SIRPα immune checkpoint in tumor immune evasion and innate immunotherapy. Life Sci (2021) 273:119150. doi: 10.1016/j.lfs.2021.119150 33662426

[156] Xia L Oyang L Lin J Tan S Han Y Wu N . The cancer metabolic reprogramming and immune response. Mol Cancer (2021) 20:28. doi: 10.1186/s12943-021-01316-8 33546704 PMC7863491

[157] DePeaux K Delgoffe GM . Metabolic barriers to cancer immunotherapy. Nat Rev Immunol (2021) 21:785–97. doi: 10.1038/s41577-021-00541-y PMC8553800 33927375

[158] Daassi D Mahoney KM Freeman GJ . The importance of exosomal PDL1 in tumour immune evasion. Nat Rev Immunol (2020) 20:209–15. doi: 10.1038/s41577-019-0264-y 31965064

[159] Wang YL Gong Y Lv Z Li L Yuan Y . Expression of PD1/PDL1 in gastric cancer at different microsatellite status and its correlation with infiltrating immune cells in the tumor microenvironment. J Cancer (2021) 12:1698–707. doi: 10.7150/jca.40500 PMC7890312 33613757

[160] Li XY Moesta AK Xiao C Nakamura K Casey M Zhang H . Targeting CD39 in cancer reveals an extracellular ATP- and inflammasome-driven tumor immunity. Cancer Discovery (2019) 9:1754–73. doi: 10.1158/2159-8290.CD-19-0541 PMC6891207 31699796

[161] Duhen T Duhen R Montler R Moses J Moudgil T de Miranda NF . Co-expression of CD39 and CD103 identifies tumor-reactive CD8 T cells in human solid tumors. Nat Commun (2018) 9:2724. doi: 10.1038/s41467-018-05072-0 30006565 PMC6045647

[162] Moesta AK Li XY Smyth MJ . Targeting CD39 in cancer. Nat Rev Immunol (2020) 20:739–55. doi: 10.1038/s41577-020-0376-4 32728220

[163] Canale FP Ramello MC Núñez N Araujo Furlan CL Bossio SN Gorosito Serrán M . CD39 expression defines cell exhaustion in tumor-infiltrating CD8(+) T cells. Cancer Res (2018) 78:115–28. doi: 10.1158/0008-5472.CAN-16-2684 29066514

[164] Neo SY Yang Y Record J Ma R Chen X Chen Z . CD73 immune checkpoint defines regulatory NK cells within the tumor microenvironment. J Clin Invest (2020) 130:1185–98. doi: 10.1172/JCI128895 PMC7269592 31770109

[165] Yasunaga M . Antibody therapeutics and immunoregulation in cancer and autoimmune disease. Semin Cancer Biol (2020) 64:1–12. doi: 10.1016/j.semcancer.2019.06.001 31181267

[166] Gou Q Dong C Xu H Khan B Jin J Liu Q . PD-L1 degradation pathway and immunotherapy for cancer. Cell Death Dis (2020) 11:955. doi: 10.1038/s41419-020-03140-2 33159034 PMC7648632

[167] Khan M Arooj S Wang H . NK cell-based immune checkpoint inhibition. Front Immunol (2020) 11:167. doi: 10.3389/fimmu.2020.00167 32117298 PMC7031489

[168] Becker PS Suck G Nowakowska P Ullrich E Seifried E Bader P . Selection and expansion of natural killer cells for NK cell-based immunotherapy. Cancer Immunol Immunother (2016) 65:477–84. doi: 10.1007/s00262-016-1792-y PMC4826432 26810567

[169] Wolf Y Anderson AC Kuchroo VK . TIM3 comes of age as an inhibitory receptor. Nat Rev Immunol (2020) 20:173–85. doi: 10.1038/s41577-019-0224-6 PMC7327798 31676858

[170] Schreiber RD Old LJ Smyth MJ . Cancer immunoediting: integrating immunity’s roles in cancer suppression and promotion. Science (2011) 331:1565–70. doi: 10.1126/science.1203486 21436444

[171] Gopalakrishnan V Helmink BA Spencer CN Reuben A Wargo JA . The influence of the gut microbiome on cancer, immunity, and cancer immunotherapy. Cancer Cell (2018) 33:570–80. doi: 10.1016/j.ccell.2018.03.015 PMC6529202 29634945

[172] Angelova M Mascaux C Galon J . Evasion before invasion: Pre-cancer immunosurveillance. Oncoimmunology (2021) 10:1912250. doi: 10.1080/2162402X.2021.1912250 33996263 PMC8078715

[173] Bates JP Derakhshandeh R Jones L Webb TJ . Mechanisms of immune evasion in breast cancer. BMC Cancer (2018) 18:556. doi: 10.1186/s12885-018-4441-3 29751789 PMC5948714

[174] Guillerey C Huntington ND Smyth MJ . Targeting natural killer cells in cancer immunotherapy. Nat Immunol (2016) 17:1025–36. doi: 10.1038/ni.3518 27540992

[175] Takeuchi Y Nishikawa H . Roles of regulatory T cells in cancer immunity. Int Immunol (2016) 28:401–9. doi: 10.1093/intimm/dxw025 PMC4986235 27160722

[176] Whiteside TL . FOXP3+ treg as a therapeutic target for promoting anti-tumor immunity. Expert Opin Ther Targets (2018) 22:353–63. doi: 10.1080/14728222.2018.1451514 PMC6126897 29532697

[177] Wu SY Fu T Jiang YZ Shao ZM . Natural killer cells in cancer biology and therapy. Mol Cancer (2020) 19:120. doi: 10.1186/s12943-020-01238-x 32762681 PMC7409673

[178] Terrén I Orrantia A Vitallé J Zenarruzabeitia O Borrego F . NK cell metabolism and tumor microenvironment. Front Immunol (2019) 10:2278. doi: 10.3389/fimmu.2019.02278 31616440 PMC6769035

[179] Hodgins JJ Khan ST Park MM Auer RC Ardolino M . Killers 2.0: NK cell therapies at the forefront of cancer control. J Clin Invest (2019) 129:3499–510. doi: 10.1172/JCI129338 PMC6715409 31478911

[180] Bui TM Wiesolek HL Sumagin R . ICAM-1: A master regulator of cellular responses in inflammation, injury resolution, and tumorigenesis. J Leukoc Biol (2020) 108:787–99. doi: 10.1002/JLB.2MR0220-549R PMC7977775 32182390

[181] Saga K Park J Nimura K Kawamura N Ishibashi A Nonomura N . NANOG helps cancer cells escape NK cell attack by downregulating ICAM1 during tumorigenesis. J Exp Clin Cancer Res (2019) 38:416. doi: 10.1186/s13046-019-1429-z 31619256 PMC6796413

[182] Smith TM Jr. Tharakan A Martin RK . Targeting ADAM10 in cancer and autoimmunity. Front Immunol (2020) 11:499. doi: 10.3389/fimmu.2020.00499 32265938 PMC7105615

[183] Tiwari A Tashiro K Dixit A Soni A Vogel K Hall B . Loss of HIF1A from pancreatic cancer cells increases expression of PPP1R1B and degradation of p53 to promote invasion and metastasis. Gastroenterology (2020) 159:1882–1897.e1885. doi: 10.1053/j.gastro.2020.07.046 32768595 PMC7680408

[184] Ou ZL Luo Z Wei W Liang S Gao TL Lu YB . Hypoxia-induced shedding of MICA and HIF1A-mediated immune escape of pancreatic cancer cells from NK cells: role of circ_0000977/miR-153 axis. RNA Biol (2019) 16:1592–603. doi: 10.1080/15476286.2019.1649585 PMC6779391 31402756

[185] Sahai E Astsaturov I Cukierman E DeNardo DG Egeblad M Evans RM . A framework for advancing our understanding of cancer-associated fibroblasts. Nat Rev Cancer (2020) 20:174–86. doi: 10.1038/s41568-019-0238-1 PMC7046529 31980749

[186] Liu T Han C Wang S Fang P Ma Z Xu L . Cancer-associated fibroblasts: an emerging target of anti-cancer immunotherapy. J Hematol Oncol (2019) 12:86. doi: 10.1186/s13045-019-0770-1 31462327 PMC6714445

[187] Nurmik M Ullmann P Rodriguez F Haan S Letellier E . In search of definitions: Cancer-associated fibroblasts and their markers. Int J Cancer (2020) 146:895–905. doi: 10.1002/ijc.32193 30734283 PMC6972582

[188] Kobayashi H Enomoto A Woods SL Burt AD Takahashi M Worthley DL . Cancer-associated fibroblasts in gastrointestinal cancer. Nat Rev Gastroenterol Hepatol (2019) 16:282–95. doi: 10.1038/s41575-019-0115-0 30778141

[189] Biffi G Tuveson DA . Diversity and biology of cancer-associated fibroblasts. Physiol Rev (2021) 101:147–76. doi: 10.1152/physrev.00048.2019 PMC7864232 32466724

[190] Chen Y McAndrews KM Kalluri R . Clinical and therapeutic relevance of cancer-associated fibroblasts. Nat Rev Clin Oncol (2021) 18:792–804. doi: 10.1038/s41571-021-00546-5 34489603 PMC8791784

[191] Liao Z Tan ZW Zhu P Tan NS . Cancer-associated fibroblasts in tumor microenvironment - accomplices in tumor malignancy. Cell Immunol (2019) 343:103729. doi: 10.1016/j.cellimm.2017.12.003 29397066

[192] Barrett RL Puré E . Cancer-associated fibroblasts and their influence on tumor immunity and immunotherapy. Elife (2020) 9:e57243. doi: 10.7554/eLife.57243 33370234 PMC7769568

[193] Gao SH Liu SZ Wang GZ Zhou GB . CXCL13 in cancer and other diseases: Biological functions, clinical significance, and therapeutic opportunities. Life (Basel) (2021) 11(12):1282. doi: 10.3390/life11121282 34947813 PMC8708574

[194] Kazanietz MG Durando M Cooke M . CXCL13 and its receptor CXCR5 in cancer: Inflammation, immune response, and beyond. Front Endocrinol (Lausanne) (2019) 10:471. doi: 10.3389/fendo.2019.00471 31354634 PMC6639976

[195] Hussain M Liu J Wang GZ Zhou GB . CXCL13 signaling in the tumor microenvironment. Adv Exp Med Biol (2021) 1302:71–90. doi: 10.1007/978-3-030-62658-7_6 34286442

[196] Chen F Pan Y Xu J Liu B Song H . Research progress of matrine’s anticancer activity and its molecular mechanism. J Ethnopharmacol (2022) 286:114914. doi: 10.1016/j.jep.2021.114914 34919987

[197] Li C Xu YH Hu YT Zhou X Huang ZS Ye JM . Matrine counteracts obesity in mice *via* inducing adipose thermogenesis by activating HSF1/PGC-1α axis. Pharmacol Res (2022) 177:106136. doi: 10.1016/j.phrs.2022.106136 35202821

[198] Xu J Hao Y Gao X Wu Y Ding Y Wang B . CircSLC7A6 promotes the progression of wilms’ tumor *via* microRNA-107/ABL proto-oncogene 2 axis. Bioengineered (2022) 13:308–18. doi: 10.1080/21655979.2021.2001204 PMC8805947 34787058

[199] Wang Y Zheng F Wang Z Lu J Zhang H . Circular RNA circ-SLC7A6 acts as a tumor suppressor in non-small cell lung cancer through abundantly sponging miR-21. Cell Cycle (2020) 19:2235–46. doi: 10.1080/15384101.2020.1806449 PMC7513860 32794418

[200] Cao Y Jiao N Sun T Ma Y Zhang X Chen H . CXCL11 correlates with antitumor immunity and an improved prognosis in colon cancer. Front Cell Dev Biol (2021) 9:646252. doi: 10.3389/fcell.2021.646252 33777950 PMC7991085

[201] Puchert M Obst J Koch C Zieger K Engele J . CXCL11 promotes tumor progression by the biased use of the chemokine receptors CXCR3 and CXCR7. Cytokine (2020) 125:154809. doi: 10.1016/j.cyto.2019.154809 31437604

[202] Tokunaga R Zhang W Naseem M Puccini A Berger MD Soni S . CXCL9, CXCL10, CXCL11/CXCR3 axis for immune activation - a target for novel cancer therapy. Cancer Treat Rev (2018) 63:40–7. doi: 10.1016/j.ctrv.2017.11.007 PMC5801162 29207310

[203] Wang D Zhao C Xu F Zhang A Jin M Zhang K . Cisplatin-resistant NSCLC cells induced by hypoxia transmit resistance to sensitive cells through exosomal PKM2. Theranostics (2021) 11:2860–75. doi: 10.7150/thno.51797 PMC7806469 33456577

[204] Cluntun AA Lukey MJ Cerione RA Locasale JW . Glutamine metabolism in cancer: Understanding the heterogeneity. Trends Cancer (2017) 3:169–80. doi: 10.1016/j.trecan.2017.01.005 PMC5383348 28393116

[205] Martínez-Reyes I Chandel NS . Cancer metabolism: Looking forward. Nat Rev Cancer (2021) 21:669–80. doi: 10.1038/s41568-021-00378-6 34272515

[206] Bader JE Voss K Rathmell JC . Targeting metabolism to improve the tumor microenvironment for cancer immunotherapy. Mol Cell (2020) 78:1019–33. doi: 10.1016/j.molcel.2020.05.034 PMC7339967 32559423

[207] Reina-Campos M Moscat J Diaz-Meco M . Metabolism shapes the tumor microenvironment. Curr Opin Cell Biol (2017) 48:47–53. doi: 10.1016/j.ceb.2017.05.006 28605656 PMC5650101

[208] Tao J Yang G Zhou W Qiu J Chen G Luo W . Targeting hypoxic tumor microenvironment in pancreatic cancer. J Hematol Oncol (2021) 14:14. doi: 10.1186/s13045-020-01030-w 33436044 PMC7805044

[209] Abdel-Wahab AF Mahmoud W Al-Harizy RM . Targeting glucose metabolism to suppress cancer progression: Prospective of anti-glycolytic cancer therapy. Pharmacol Res (2019) 150:104511. doi: 10.1016/j.phrs.2019.104511 31678210

[210] Ghanavat M Shahrouzian M Deris Zayeri Z Banihashemi S Kazemi SM Saki N . Digging deeper through glucose metabolism and its regulators in cancer and metastasis. Life Sci (2021) 264:118603. doi: 10.1016/j.lfs.2020.118603 33091446

[211] Xu G Li M Wu J Qin C Tao Y He H . Circular RNA circNRIP1 sponges microRNA-138-5p to maintain hypoxia-induced resistance to 5-fluorouracil through HIF-1α-Dependent glucose metabolism in gastric carcinoma. Cancer Manag Res (2020) 12:2789–802. doi: 10.2147/CMAR.S246272 PMC7186590 32425596

[212] Marcucci F Rumio C . Glycolysis-induced drug resistance in tumors-a response to danger signals? Neoplasia (2021) 23:234–45. doi: 10.1016/j.neo.2020.12.009 PMC7804361 33418276

[213] Zhu S Guo Y Zhang X Liu H Yin M Chen X . Pyruvate kinase M2 (PKM2) in cancer and cancer therapeutics. Cancer Lett (2021) 503:240–8. doi: 10.1016/j.canlet.2020.11.018 33246091

[214] Anderson PM Lalla RV . Glutamine for amelioration of radiation and chemotherapy associated mucositis during cancer therapy. Nutrients (2020) 12(6):1675. doi: 10.3390/nu12061675 PMC7352314 32512833

[215] Cruzat V Macedo Rogero M Noel Keane K Curi R Newsholme P . Glutamine: Metabolism and immune function, supplementation and clinical translation. Nutrients (2018) 10(11):1564. doi: 10.3390/nu10111564 PMC6266414 30360490

[216] Kim MH Kim H . The roles of glutamine in the intestine and its implication in intestinal diseases. Int J Mol Sci (2017) 18(5):1051. doi: 10.3390/ijms18051051 PMC5454963 28498331

[217] Masisi BK El Ansari R Alfarsi L Rakha EA Green AR Craze ML . The role of glutaminase in cancer. Histopathology (2020) 76:498–508. doi: 10.1111/his.14014 31596504

[218] Matés JM Campos-Sandoval JA Márquez J . Glutaminase isoenzymes in the metabolic therapy of cancer. Biochim Biophys Acta Rev Cancer (2018) 1870:158–64. doi: 10.1016/j.bbcan.2018.07.007 30053497

[219] Zhang J Pavlova NN Thompson CB . Cancer cell metabolism: the essential role of the nonessential amino acid, glutamine. EMBO J (2017) 36:1302–15. doi: 10.15252/embj.201696151 PMC5430235 28420743

[220] Amelio I Cutruzzolá F Antonov A Agostini M Melino G . Serine and glycine metabolism in cancer. Trends Biochem Sci (2014) 39:191–8. doi: 10.1016/j.tibs.2014.02.004 PMC3989988 24657017

[221] Pan S Fan M Liu Z Li X Wang H . Serine, glycine and one−carbon metabolism in cancer (Review). Int J Oncol (2021) 58:158–70. doi: 10.3892/ijo.2020.5158 PMC7864012 33491748

[222] Newman AC Maddocks ODK . One-carbon metabolism in cancer. Br J Cancer (2017) 116:1499–504. doi: 10.1038/bjc.2017.118 PMC5518849 28472819

[223] Li AM Ye J . Reprogramming of serine, glycine and one-carbon metabolism in cancer. Biochim Biophys Acta Mol Basis Dis (2020) 1866:165841. doi: 10.1016/j.bbadis.2020.165841 32439610 PMC7442608

[224] Zhao JY Feng KR Wang F Zhang JW Cheng JF Lin GQ . A retrospective overview of PHGDH and its inhibitors for regulating cancer metabolism. Eur J Med Chem (2021) 217:113379. doi: 10.1016/j.ejmech.2021.113379 33756126

[225] Ou Y Wang SJ Jiang L Zheng B Gu W . p53 protein-mediated regulation of phosphoglycerate dehydrogenase (PHGDH) is crucial for the apoptotic response upon serine starvation. J Biol Chem (2015) 290:457–66. doi: 10.1074/jbc.M114.616359 PMC4281747 25404730

[226] Zhao L Guo Y Guo Y Ji X Fan D Chen C . Effect and mechanism of circRNAs in tumor angiogenesis and clinical application. Int J Cancer (2022) 150:1223–32. doi: 10.1002/ijc.33863 34724210

[227] Jiang X Wang J Deng X Xiong F Zhang S Gong Z . The role of microenvironment in tumor angiogenesis. J Exp Clin Cancer Res (2020) 39:204. doi: 10.1186/s13046-020-01709-5 32993787 PMC7526376

[228] Kretschmer M Rüdiger D Zahler S . Mechanical aspects of angiogenesis. Cancers (Basel) (2021) 13(19):4987. doi: 10.3390/cancers13194987 34638470 PMC8508205

[229] Zeng Y Fu BM . Resistance mechanisms of anti-angiogenic therapy and exosomes-mediated revascularization in cancer. Front Cell Dev Biol (2020) 8:610661. doi: 10.3389/fcell.2020.610661 33363174 PMC7755714

[230] Schultz CW Preet R Dhir T Dixon DA Brody JR . Understanding and targeting the disease-related RNA binding protein human antigen r (HuR). Wiley Interdiscip Rev RNA (2020) 11:e1581. doi: 10.1002/wrna.1581 31970930 PMC7482136

[231] Karaman S Detmar M . Mechanisms of lymphatic metastasis. J Clin Invest (2014) 124:922–8. doi: 10.1172/JCI71606 PMC3938272 24590277

[232] Kong D Zhou H Neelakantan D Hughes CJ Hsu JY Srinivasan RR . VEGF-c mediates tumor growth and metastasis through promoting EMT-epithelial breast cancer cell crosstalk. Oncogene (2021) 40:964–79. doi: 10.1038/s41388-020-01539-x PMC7867573 33299122

[233] Zheng H Chen C Luo Y Yu M He W An M . Tumor-derived exosomal BCYRN1 activates WNT5A/VEGF-C/VEGFR3 feedforward loop to drive lymphatic metastasis of bladder cancer. Clin Transl Med (2021) 11:e497. doi: 10.1002/ctm2.497 34323412 PMC8288020

[234] Lin QY Zhang YL Bai J Liu JQ Li HH . VEGF-C/VEGFR-3 axis protects against pressure-overload induced cardiac dysfunction through regulation of lymphangiogenesis. Clin Transl Med (2021) 11:e374. doi: 10.1002/ctm2.374 33783987 PMC7989711

[235] Vallejo-Díaz J Chagoyen M Olazabal-Morán M González-García A Carrera AC . The opposing roles of PIK3R1/p85α and PIK3R2/p85β in cancer. Trends Cancer (2019) 5:233–44. doi: 10.1016/j.trecan.2019.02.009 30961830

[236] Cottrell CE Bender NR Zimmermann MT Heusel JW Corliss M Evenson MJ . Somatic PIK3R1 variation as a cause of vascular malformations and overgrowth. Genet Med (2021) 23:1882–8. doi: 10.1038/s41436-021-01211-z PMC8486672 34040190

[237] Vaupel P Multhoff G . Hypoxia-/HIF-1α-Driven factors of the tumor microenvironment impeding antitumor immune responses and promoting malignant progression. Adv Exp Med Biol (2018) 1072:171–5. doi: 10.1007/978-3-319-91287-5_27 30178341

[238] Riera-Domingo C Audigé A Granja S Cheng WC Ho PC Baltazar F . Immunity, hypoxia, and metabolism-the ménage à trois of cancer: Implications for immunotherapy. Physiol Rev (2020) 100:1–102. doi: 10.1152/physrev.00018.2019 31414610

[239] de Heer EC Jalving M Harris AL . HIFs, angiogenesis, and metabolism: elusive enemies in breast cancer. J Clin Invest (2020) 130:5074–87. doi: 10.1172/JCI137552 PMC7524491 32870818

[240] Jing X Yang F Shao C Wei K Xie M Shen H . Role of hypoxia in cancer therapy by regulating the tumor microenvironment. Mol Cancer (2019) 18:157. doi: 10.1186/s12943-019-1089-9 31711497 PMC6844052

[241] Nho RS Peterson M . Eukaryotic translation initiation factor 4E binding protein 1 (4EBP-1) function is suppressed by src and protein phosphatase 2A (PP2A) on extracellular matrix. J Biol Chem (2011) 286:31953–65. doi: 10.1074/jbc.M111.222299 PMC3173218 21784851

[242] Zhang J Zhang Q . VHL and hypoxia signaling: Beyond HIF in cancer. Biomedicines (2018) 6. doi: 10.3390/biomedicines6010035 PMC5874692 29562667

[243] Bhattacharya S Calar K Evans C Petrasko M de la Puente P . Bioengineering the oxygen-deprived tumor microenvironment within a three-dimensional platform for studying tumor-immune interactions. Front Bioeng Biotechnol (2020) 8:1040. doi: 10.3389/fbioe.2020.01040 33015012 PMC7498579

[244] Brabletz S Schuhwerk H Brabletz T Stemmler MP . Dynamic EMT: A multi-tool for tumor progression. EMBO J (2021) 40:e108647. doi: 10.15252/embj.2021108647 34459003 PMC8441439

[245] Bakir B Chiarella AM Pitarresi JR Rustgi AK . EMT, MET, plasticity, and tumor metastasis. Trends Cell Biol (2020) 30:764–76. doi: 10.1016/j.tcb.2020.07.003 PMC7647095 32800658

[246] De Las Rivas J Brozovic A Izraely S Casas-Pais A Witz IP Figueroa A . Cancer drug resistance induced by EMT: novel therapeutic strategies. Arch Toxicol (2021) 95:2279–97. doi: 10.1007/s00204-021-03063-7 PMC8241801 34003341

[247] Li J Zhang G Liu CG Xiang X Le MTN Sethi G . The potential role of exosomal circRNAs in the tumor microenvironment: Insights into cancer diagnosis and therapy. Theranostics (2022) 12:87–104. doi: 10.7150/thno.64096 34987636 PMC8690929

[248] Wu X Xin Z Zou Z Lu C Yu Z Feng S . SRY-related high-mobility-group box 4: Crucial regulators of the EMT in cancer. Semin Cancer Biol (2020) 67:114–21. doi: 10.1016/j.semcancer.2019.06.008 31199986

[249] Wilson MM Weinberg RA Lees JA Guen VJ . Emerging mechanisms by which EMT programs control stemness. Trends Cancer (2020) 6:775–80. doi: 10.1016/j.trecan.2020.03.011 32312682

[250] Mohan V Das A Sagi I . Emerging roles of ECM remodeling processes in cancer. Semin Cancer Biol (2020) 62:192–200. doi: 10.1016/j.semcancer.2019.09.004 31518697

[251] Roma-Rodrigues C Mendes R Baptista PV Fernandes AR . Targeting tumor microenvironment for cancer therapy. Int J Mol Sci (2019) 20(4):840. doi: 10.3390/ijms20040840 PMC6413095 30781344

[252] Zeltz C Primac I Erusappan P Alam J Noel A Gullberg D . Cancer-associated fibroblasts in desmoplastic tumors: emerging role of integrins. Semin Cancer Biol (2020) 62:166–81. doi: 10.1016/j.semcancer.2019.08.004 31415910

[253] Huang H . Matrix metalloproteinase-9 (MMP-9) as a cancer biomarker and MMP-9 biosensors: Recent advances. Sensors (Basel) (2018) 18(10):3249. doi: 10.3390/s18103249 PMC6211011 30262739

[254] Najafi M Farhood B Mortezaee K . Extracellular matrix (ECM) stiffness and degradation as cancer drivers. J Cell Biochem (2019) 120:2782–90. doi: 10.1002/jcb.27681 30321449

[255] Nissen NI Karsdal M Willumsen N . Collagens and cancer associated fibroblasts in the reactive stroma and its relation to cancer biology. J Exp Clin Cancer Res (2019) 38:115. doi: 10.1186/s13046-019-1110-6 30841909 PMC6404286

[256] Lyu Y Xiao Q Yin L Yang L He W . Potent delivery of an MMP inhibitor to the tumor microenvironment with thermosensitive liposomes for the suppression of metastasis and angiogenesis. Signal Transduct Target Ther (2019) 4:26. doi: 10.1038/s41392-019-0054-9 31637006 PMC6799847

[257] Niland S Eble JA . Hold on or cut? integrin- and MMP-mediated cell-matrix interactions in the tumor microenvironmentB. Int J Mol Sci (2020) 22(1):238. doi: 10.3390/ijms22010238 PMC7794804 33379400

[258] Maddineni P Kasetti RB Kodati B Yacoub S Zode GS . Sodium 4-phenylbutyrate reduces ocular hypertension by degrading extracellular matrix deposition. Via Activation MMP9 Int J Mol Sci (2021) 22(18):10095. doi: 10.3390/ijms221810095 34576258 PMC8465971

[259] Walter L Canup B Pujada A Bui TA Arbasi B Laroui H . Matrix metalloproteinase 9 (MMP9) limits reactive oxygen species (ROS) accumulation and DNA damage in colitis-associated cancer. Cell Death Dis (2020) 11:767. doi: 10.1038/s41419-020-02959-z 32943603 PMC7498454

[260] Pittayapruek P Meephansan J Prapapan O Komine M Ohtsuki M . Role of matrix metalloproteinases in photoaging and photocarcinogenesis. Int J Mol Sci (2016) 17(6):868. doi: 10.3390/ijms17060868 PMC4926402 27271600

[261] Zhang Q Wang W Zhou Q Chen C Yuan W Liu J . Roles of circRNAs in the tumour microenvironment. Mol Cancer (2020) 19:14. doi: 10.1186/s12943-019-1125-9 31973726 PMC6977266

[262] Carlos-Reyes Á Romero-Garcia S Contreras-Sanzón E Ruiz V Prado-Garcia H . Role of circular RNAs in the regulation of immune cells in response to cancer therapies. Front Genet (2022) 13:823238. doi: 10.3389/fgene.2022.823238 35186039 PMC8847670

[263] Bukowski K Kciuk M Kontek R . Mechanisms of multidrug resistance in cancer chemotherapy. Int J Mol Sci (2020) 21(9):3233. doi: 10.3390/ijms21093233 PMC7247559 32370233

[264] Rizzuti IF Mascheroni P Arcucci S Ben-Mériem Z Prunet A Barentin C . Mechanical control of cell proliferation increases resistance to chemotherapeutic agents. Phys Rev Lett (2020) 125:128103. doi: 10.1103/PhysRevLett.125.128103 33016731

[265] Newton PK Ma Y . Nonlinear adaptive control of competitive release and chemotherapeutic resistance. Phys Rev E (2019) 99:022404. doi: 10.1103/PhysRevE.99.022404 30934318 PMC7515604

[266] Cui C Yang J Li X Liu D Fu L Wang X . Functions and mechanisms of circular RNAs in cancer radiotherapy and chemotherapy resistance. Mol Cancer (2020) 19:58. doi: 10.1186/s12943-020-01180-y 32171304 PMC7071709

[267] Lowrence RC Subramaniapillai SG Ulaganathan V Nagarajan S . Tackling drug resistance with efflux pump inhibitors: From bacteria to cancerous cells. Crit Rev Microbiol (2019) 45:334–53. doi: 10.1080/1040841X.2019.1607248 31248314

[268] Robey RW Pluchino KM Hall MD Fojo AT Bates SE Gottesman MM . Revisiting the role of ABC transporters in multidrug-resistant cancer. Nat Rev Cancer (2018) 18:452–64. doi: 10.1038/s41568-018-0005-8 PMC6622180 29643473

[269] Onorati AV Dyczynski M Ojha R Amaravadi RK . Targeting autophagy in cancer. Cancer (2018) 124:3307–18. doi: 10.1002/cncr.31335 PMC6108917 29671878

[270] Li X He S Ma B . Autophagy and autophagy-related proteins in cancer. Mol Cancer (2020) 19:12. doi: 10.1186/s12943-020-1138-4 31969156 PMC6975070

[271] Kocaturk NM Akkoc Y Kig C Bayraktar O Gozuacik D Kutlu O . Autophagy as a molecular target for cancer treatment. Eur J Pharm Sci (2019) 134:116–37. doi: 10.1016/j.ejps.2019.04.011 30981885

[272] Russo M Russo GL . Autophagy inducers in cancer. Biochem Pharmacol (2018) 153:51–61. doi: 10.1016/j.bcp.2018.02.007 29438677

[273] Yamazaki T Bravo-San Pedro JM Galluzzi L Kroemer G Pietrocola F . Autophagy in the cancer-immunity dialogue. Adv Drug Delivery Rev (2021) 169:40–50. doi: 10.1016/j.addr.2020.12.003 33301821

[274] Zamame Ramirez JA Romagnoli GG Kaneno R . Inhibiting autophagy to prevent drug resistance and improve anti-tumor therapy. Life Sci (2021) 265:118745. doi: 10.1016/j.lfs.2020.118745 33186569

[275] Ferro F Servais S Besson P Roger S Dumas JF Brisson L . Autophagy and mitophagy in cancer metabolic remodelling. Semin Cell Dev Biol (2020) 98:129–38. doi: 10.1016/j.semcdb.2019.05.029 31154012

[276] Liang G Ling Y Mehrpour M Saw PE Liu Z Tan W . Autophagy-associated circRNA circCDYL augments autophagy and promotes breast cancer progression. Mol Cancer (2020) 19:65. doi: 10.1186/s12943-020-01152-2 32213200 PMC7093993

[277] Mohammad RM Muqbil I Lowe L Yedjou C Hsu HY Lin LT . Broad targeting of resistance to apoptosis in cancer. Semin Cancer Biol (2015) 35 Suppl:S78–s103. doi: 10.1016/j.semcancer.2015.03.001 25936818 PMC4720504

[278] Li R Jiang J Shi H Qian H Zhang X Xu W . CircRNA: a rising star in gastric cancer. Cell Mol Life Sci (2020) 77:1661–80. doi: 10.1007/s00018-019-03345-5 PMC11104848 31659415

[279] Ma B Ran R Liao HY Zhang HH . The paradoxical role of matrix metalloproteinase-11 in cancer. BioMed Pharmacother (2021) 141:111899. doi: 10.1016/j.biopha.2021.111899 34346316

[280] Leung HW Leung CON Lau EY Chung KPS Mok EH Lei MML . EPHB2 activates β-catenin to enhance cancer stem cell properties and drive sorafenib resistance in hepatocellular carcinoma. Cancer Res (2021) 81:3229–40. doi: 10.1158/0008-5472.CAN-21-0184 33903122

[281] Wang Y Liu J Ma J Sun T Zhou Q Wang W . Exosomal circRNAs: biogenesis, effect and application in human diseases. Mol Cancer (2019) 18:116. doi: 10.1186/s12943-019-1041-z 31277663 PMC6610963

[282] Zhang Y Liu Q Zhang X Huang H Tang S Chai Y . Recent advances in exosome-mediated nucleic acid delivery for cancer therapy. J Nanobiotech (2022) 20:279. doi: 10.1186/s12951-022-01472-z PMC9194774 35701788

[283] Zhou ZW Zheng W Xiang Z Ye CS Yin QQ Wang SH . Clinical implications of exosome-derived noncoding RNAs in liver. Lab Invest (2022) 102:464–73. doi: 10.1038/s41374-021-00723-1 35013531

[284] Huang XY Huang ZL Huang J Xu B Huang XY Xu YH . Exosomal circRNA-100338 promotes hepatocellular carcinoma metastasis *via* enhancing invasiveness and angiogenesis. J Exp Clin Cancer Res (2020) 39:20. doi: 10.1186/s13046-020-1529-9 31973767 PMC6979009

[285] Chen S Chen Z Li Z Li S Wen Z Cao L . Tumor-associated macrophages promote cholangiocarcinoma progression *via* exosomal Circ_0020256. Cell Death Dis (2022) 13:94. doi: 10.1038/s41419-022-04534-0 35091535 PMC8799724

[286] Sang H Zhang W Peng L Wei S Zhu X Huang K . Exosomal circRELL1 serves as a miR-637 sponge to modulate gastric cancer progression *via* regulating autophagy activation. Cell Death Dis (2022) 13:56. doi: 10.1038/s41419-021-04364-6 35027539 PMC8758736

[287] Chen W Quan Y Fan S Wang H Liang J Huang L . Exosome-transmitted circular RNA hsa_circ_0051443 suppresses hepatocellular carcinoma progression. Cancer Lett (2020) 475:119–28. doi: 10.1016/j.canlet.2020.01.022 32014458

[288] Li Y Zheng Q Bao C Li S Guo W Zhao J . Circular RNA is enriched and stable in exosomes: a promising biomarker for cancer diagnosis. Cell Res (2015) 25:981–4. doi: 10.1038/cr.2015.82 PMC4528056 26138677

[289] Xu Y Kong S Qin S Shen X Ju S . Exosomal circRNAs: Sorting mechanisms, roles and clinical applications in tumors. Front Cell Dev Biol (2020) 8:581558. doi: 10.3389/fcell.2020.581558 33324638 PMC7723975

[290] Zheng R Zhang K Tan S Gao F Zhang Y Xu W . Exosomal circLPAR1 functions in colorectal cancer diagnosis and tumorigenesis through suppressing BRD4 *via* METTL3-eIF3h interaction. Mol Cancer (2022) 21:49. doi: 10.1186/s12943-021-01471-y 35164758 PMC8842935

[291] Zheng P Gao H Xie X Lu P . Plasma exosomal hsa_circ_0015286 as a potential diagnostic and prognostic biomarker for gastric cancer. Pathol Oncol Res (2022) 28:1610446. doi: 10.3389/pore.2022.1610446 35755416 PMC9218071

[292] Luo Y Liu F Gui R . High expression of circulating exosomal circAKT3 is associated with higher recurrence in HCC patients undergoing surgical treatment. Surg Oncol (2020) 33:276–81. doi: 10.1016/j.suronc.2020.04.021 32561093

